# Exosome enrichment from human serum using polyethylene glycol precipitation

**DOI:** 10.1186/s40543-025-00521-0

**Published:** 2025-12-23

**Authors:** Reham M. Marzouk, Mohamed A. Gab-Allah, Hyojin Hwang, Maryam Adelipour, Hyeongyu Yu, Ga Seul Lee, Jeong Hee Moon, David M. Lubman, Jeongkwon Kim

**Affiliations:** 1 Department of Chemistry, Chungnam National University, Daejeon, Republic of Korea; 2 Organic Analysis Laboratory, National Institute of Standards, Tersa St., Al-Haram, P. O. Box: 136, Giza, 12211 Egypt; 3 Department of Clinical Biochemistry, School of Medicine, Ahvaz Jundishapur University of Medical Sciences, Ahvaz, Iran; 4 Research Facility and Analysis Center, Korea Research Institute of Bioscience and Biotechnology, Daejeon, Republic of Korea; 5 Department of Surgery, University of Michigan Medical Center, Ann Arbor, MI USA

**Keywords:** Extracellular vesicles, Exosomes, Polyethylene glycol precipitation, ExoQuick, Size exclusion chromatography, LC-MS/MS

## Abstract

Exosomes, nanosized extracellular vesicles that play key roles in intercellular communication, have great potential in diagnostic and therapeutic applications. However, isolating exosomes from complex biological matrices such as human serum remains challenging due to limitations in scalability, cost, and purity of current methods. In this study, we developed and optimized a polyethylene glycol (PEG) precipitation method as a cost-effective alternative to the commercial ExoQuick kit for efficient exosome enrichment from pretreated human serum. The efficiency and reproducibility of PEG precipitation were systematically evaluated and compared with ExoQuick using various analytical techniques, including size exclusion chromatography (SEC), scanning electron microscopy (SEM), nanoparticle tracking analysis (NTA), Western blotting, and nano-liquid chromatography–tandem mass spectrometry (nano-LC–MS/MS). SEC confirmed comparable exosome recovery between PEG and ExoQuick, while SEM and NTA analyses verified intact morphology and consistent size distribution of exosomes. Western blotting confirmed the presence of characteristic exosomal markers (CD63 and CD9) and a reduction of contaminating proteins such as albumin and apolipoprotein A1. Moreover, proteomic analysis by nano-LC–MS/MS identified diverse exosomal proteins, confirming the isolation of intact exosomes. Repeated PEG precipitation up to four cycles further improved exosome purity without compromising yield. These results demonstrate that PEG precipitation is a scalable, accessible, and cost-effective method that provides comparable exosome enrichment efficiency to ExoQuick. The optimized protocol offers a practical solution for analytical laboratories to enable cost-effective exosome-based studies in diagnostics and therapeutics.

**Supplementary Information:**

The online version contains supplementary material available at 10.1186/s40543-025-00521-0.

## Introduction

Exosomes are phospholipid bilayer-enclosed biological nanoparticles with sizes ranging from 30 to 200 nanometers (Onozato et al. 2018). These cell-derived vesicles represent a specific subtype of small extracellular vesicles (EVs) released by all living cells and can be found in various biological fluids, including blood, urine, saliva, breast milk, and bile, among others (Dear et al. 2013; Gao et al. 2019b; Lau et al., 2012). Exosomes contain various biomolecules, such as deoxyribonucleic acid (DNA), messenger ribonucleic acid (mRNA), or microRNAs, as well as lipids, proteins, and other small molecules (Lau et al., 2012; Yamada et al. 2012). Exosomes have attracted much scientific interest since they play crucial roles in numerous physiological and pathological processes, including cancer, inflammation, signal transduction, immune responses, and are intercellular communication carriers for proteins, nucleic acids, and other biomolecules (Bellingham et al. 2012; Fleming et al. 2014; Ha et al., 2016; Schorey et al., 2008).

Additionally, the use of exosomes associated with malignancies provides a non-invasive method for monitoring tumor growth, drug resistance, and metastasis (Gao et al. 2021; Vasconcelos et al. 2019), where exosomes are being investigated as potential biomarkers in disease diagnosis. More recently, a novel aptasensor-based microfluidic chip with a microelectrode sensor has been developed for the efficient enrichment and sensitive, label-free detection of tumor-derived exosomes (TEX) using electrochemical impedance spectroscopy, demonstrating high selectivity, stability, and clinical diagnostic potential (Li et al. 2024a). There is also an increasing interest in utilizing their potential as excellent drug delivery carriers (Aheget et al. 2020; Amiri et al. 2021). This can be attributed to the ability of exosomes to transfer and exchange their cargo between bodily fluids and cells while maintaining their intact vesicle structure, which protects the enclosed materials from enzymatic hydrolysis and degradation (Gao et al. 2019a). On the other hand, capillary electrophoresis (CE) has also been explored as a method for EV characterization, offering a fluorescence-based approach to identify and analyze EV subpopulations, despite challenges related to standard availability and signal confirmation (Steć et al. 2024). Recently, quantitative analysis of exosomes is gaining popularity and has been achieved indirectly using graphene oxide adsorbed with horseradish peroxidase (Li et al. 2024b).

Enriching and isolating exosomes from biological samples is crucial for their subsequent analysis. However, this process is particularly challenging for serum or plasma due to the typically small sample volume, high viscosity, elevated protein concentration, and the presence of other contaminating particles, particularly lipoproteins, which have diameters similar to exosomes (Macías et al. 2019). Despite the growing interest in understanding the function and composition of exosomes, standardized methods for their isolation and purification are still lacking. At present, various methodologies have been available to enrich exosomes from various body fluids such as serum (Kim et al. 2015), plasma, urine (Park et al. 2023), saliva (Lau et al., 2012; Sun et al. 2017), and other fluids (Hu et al. 2022; Vaswani et al. 2019). The most commonly used exosome enrichment method is ultracentrifugation (UC), which is often combined with sucrose density gradients and yields highly pure exosomes (Gupta et al. 2018). However, this approach necessitates a lengthy separation time (up to 8–10 h) with multiple high-speed centrifugation steps, which limits its application (Jalaludin et al. 2023a; Taylor et al., 2015; Théry et al. 2006). Moreover, other concerns regarding the effectiveness of UC-based approaches have been raised due to potential exosome damage attributed to the high shear forces involved (Ding et al. 2018; Witwer et al. 2013).

Immunoaffinity techniques have facilitated the isolation of exosomes by targeting distinctive markers like CD63, CD81, and CD9 (Greening et al. 2015; Mathivanan et al. 2010; Melo et al. 2015; Zhu et al. 2021). Nonetheless, these approaches often encounter challenges, including prolonged processing times (4–20 h), limited reproducibility, high cost, and lower exosome yield. Exosome isolation kits from different producers have been launched, allowing for their rapid isolation from complex biological samples (Ding et al. 2018; Li et al. 2017); however, these kits are expensive, thereby limiting their use in large-scale applications. Furthermore, various techniques have been proposed for the isolation and purification of exosomes based on size exclusion chromatography (SEC), ultrafiltration, dialysis, and microfluidics-based platforms (El Ouahabi et al. 2021; Helwa et al. 2017; Li et al. 2017; Macías et al. 2019; Nakai et al. 2016).

Recently, alternative approaches such as hydrophobic interaction chromatography using capillary-channeled polymer fiber columns have been developed, offering a rapid (15-min) and cost-effective exosome isolation method with high-quality particle concentration and sizing information (Wysor et al., 2024). The EXODUS method has also emerged as a promising alternative, offering higher exosome yield and purity compared to conventional methods (Chen et al. 2021; Ni et al. 2024). While these approaches have potential for specific applications, they are still restricted to low throughput and often need extensive instrumentation (Böing et al. 2014). Therefore, there is a real need for a quick, easy, cost-effective, and efficient isolation method to enable routine exosome analysis.

Polymeric precipitation techniques are particularly promising since they can provide a much faster and easier approach to exosome isolation (Stam et al. 2021; Weng et al. 2016). Among these techniques, polyethylene glycol (PEG) precipitation is an emerging method that allows straightforward, low-cost, reproducible, and effective isolation of exosomes from a small sample volume, without requiring sophisticated equipment (Marzouk et al. 2025; Rider et al. 2016). This method relies on the polymer’s ability to aggregate particles, facilitating their precipitation from solution. PEG is a water-soluble polymer that can dehydrate the vesicles and facilitate the precipitation of exosomes from biological fluids, allowing their precipitation using low-speed centrifugation (Weng et al. 2016). Several studies have explored the use of PEG of different average molecular weights and concentrations for exosome enrichment (Ludwig et al. 2018; Rider et al. 2016; Stam et al. 2021; Weng et al. 2016). The PEG precipitation method stands out for its simplicity, cost-effectiveness, and scalability, making it an attractive choice for large-scale applications (Rider et al. 2016).

In the current investigation, we evaluated the PEG precipitation method for exosome isolation and purification from human serum, comparing its efficacy with that of the commercially available and widely used ExoQuick kit. PEG precipitation involved the addition of PEG to the pretreated serum, followed by incubation at low temperatures, and subsequent centrifugation at a low speed to pellet the exosomes. The process was repeated up to four enrichment cycles to enhance exosome purity, and the final exosome pellet was resuspended in a buffer solution for further analysis. The presence and purity of the isolated exosomes were confirmed using various analytical techniques, including size-exclusion chromatography (SEC), scanning electron microscopy (SEM), nanoparticle tracking analysis (NTA), Western blotting, and nano-LC-MS/MS. These results highlight PEG precipitation as a viable, cost-effective alternative for exosome isolation, offering performance equivalent to established commercial methods.

## Materials and methods

### Chemicals and reagents

Pooled normal human serum samples were obtained from Innovative Research (Novi, MI, USA). Phosphate-buffered saline (PBS), polyethylene glycol (PEG) with an average molecular weight of 10,000 Da (PEG 10,000), triethylammonium bicarbonate (TEAB), iodoacetamide (IAA), α-cyano-4-hydroxycinnamic acid (CHCA), human serum albumin, and human serum immunoglobulin G were provided by Sigma-Aldrich (Steinheim, Germany). A 0.45 μm membrane filter was purchased from Hyundai Micro (Gyeonggi-do, Korea). 5× lane marker non-reducing sample buffer was acquired from Thermo Scientific, Waltham, MA, USA (catalog number: 39001). Trifluoroacetic acid was provided by Samchun Pure Chemical Co. Ltd. (Anyang, Korea), and acetonitrile was supplied by Duksan Pure Chemicals (Ansan, Korea). ExoQuick kits were purchased from System Biosciences (SBI, Palo Alto, CA, USA). Ultrahydrogel 1000 Column (7.8 mm ×300 mm, 1000 Å) used as the size exclusion column was provided by Waters (Milford, USA). PVDF membrane (Bio-Rad, catalog number: 162–0177). Anti-CD63 antibody (ab59479, Mouse monoclonal to CD63), goat anti-mouse IgG H&L (horseradish peroxidase) preadsorbed (ab97040) were from Abcam (Cambridge, MA). Anti-CD9 antibody (CD9 Monoclonal Antibody (Ts9)) was from Thermo Fisher Scientific Baltics UAB (Vilnius, Lithuania). Anti-albumin, Anti-Apolipoprotein A1 Rabbit mab, and Goat anti-rabbit IgG H&L (horseradish peroxidase) preadsorbed were provided by ABclonal technology (Massachusetts, USA).

### Serum pretreatment

To remove cells and other large particles, the serum was first diluted with an equal volume of PBS (1:1 ratio). This diluted serum was then subjected to low-speed centrifugation at 2,000 × g for 30 min at 4 °C to pellet the larger particulates and cells. The supernatant from this initial centrifugation was carefully collected and centrifuged at 12,000 × g for 45 min at 4 °C to eliminate smaller debris and any remaining cells. The resulting supernatant was then passed through a 0.45 μm filter to ensure the removal of any residual cell particles or debris, resulting in a clear solution of pretreated serum ready for exosome enrichment. Consequently, 1 mL of human serum resulted in approximately 1.7 mL of pretreated serum solution.

### Exosome enrichment using PEG precipitation

**Fig. 1:**
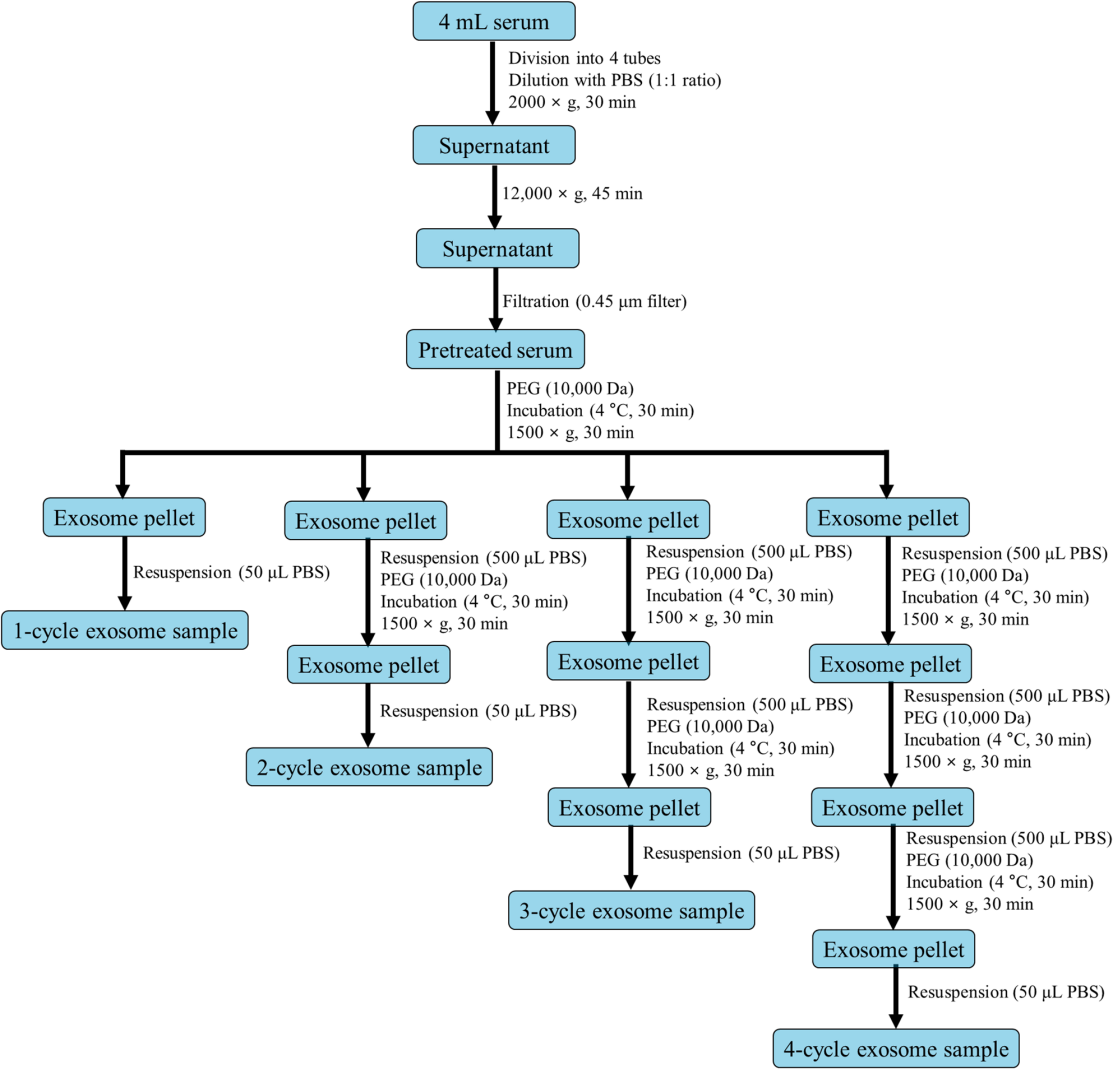
The analytical scheme for exosome enrichment from human serum using the PEG precipitation technique. The exosome fractions were obtained through multiple cycles of precipitation, ranging from 1 to 4 cycles of PEG treatment, applied to pretreated serum samples (1 mL each)

The PEG solution was prepared by dissolving 1 g of solid PEG 10,000 in 2 mL of PBS with sonication to enhance the PEG solubility, resulting in a total volume of 3 mL and a 33 (w/v)% PEG solution. For the PEG precipitation of the exosomes in serum, all of the pretreated serum (approximately 1.7 mL) was treated with the PEG solution in a mixing ratio of 9:1 (v/v) by adding 189 µL of the PEG solution, resulting in a 3.3 (w/v)% PEG concentration. In the initial stages of the development of this method, other serum-to-PEG ratios such as 18:1, 4:1, 2:1, and 1:1 (v/v), equivalent to PEG percentages of 1.7, 6.6, 11.0, and 16.5 (w/v)%, respectively, along with different molecular weights of PEG (8000, 12000, 20000, and 35000 Da) were also investigated. The mixture was gently mixed and incubated at 4 °C for 30 min, followed by a subsequent centrifugation at 1500 × g for 30 min at 4 °C to form an exosome pellet. The supernatant was discarded, and the remaining pellet was resuspended in 50 µL of PBS for further analysis. To enhance the efficiency of exosome enrichment, the procedure was repeated up to four times, with the pellet resuspended in 500 µL of PBS for PEG precipitation instead of 50 µL, and PEG added to maintain the same ratio as in the first cycle. Each cycle involved the serum treatment with PEG, incubation, centrifugation, and resuspension of the pellet in PBS. As mentioned above, the final exosome pellet, after each enrichment cycle, was resuspended in 50 µL of PBS and stored at 4 °C until further use. The detailed analytical scheme for exosome enrichment from human serum using PEG is illustrated in Fig. 1.

### Exosome enrichment using exoquick kit

Enrichment of exosomes using ExoQuick buffer was performed according to the manufacturer’s instructions. Briefly, 1.7 mL of pretreated serum was gently mixed with 214 µL ExoQuick solution and incubated at 4 °C for 30 min. Subsequently, the sample was centrifuged at 1500 × g for 30 min at 4 °C to form an exosome pellet. The supernatant was discarded, and the resulting exosome pellet was resuspended in 50 µL of PBS. To enhance the efficiency of exosome enrichment, this procedure was repeated up to four times, similar to the PEG enrichment method. Each cycle involved treating the serum with ExoQuick solution, incubating, centrifuging, and resuspending the pellet in PBS. The final exosome pellet, after each cycle of enrichment, was resuspended in 50 µL of PBS and stored at 4 °C until further use.

### Size exclusion chromatography

The separation of protein and exosome peaks was carried out using size exclusion chromatography (SEC, Water e2695) (CNU Chemistry Core Facility, Daejeon, South Korea) on an Ultrahydrogel 1000 Column (7.8 × 300 mm, part No. WAT011535) with a mobile phase consisting of PBS solution. To remove large particles from the PBS solution, it was filtered using a membrane filter with a 0.22 μm pore size before its use. The detection of exosome samples was conducted in two stages using a photodiode array detector (Waters 2998) and a UV-visible detector (Waters 2489). The entire analysis was conducted at a constant column temperature of 25 °C and a flow rate of 1 mL/min with a sample injection volume of 10 µL. Before each sample injection, the SEC system was allowed to stabilize until the system pressure decreased below 450 psi.

### Matrix-assisted laser desorption/ionization time-of-flight mass spectrometry (MALDI-TOF MS)

For MALDI-TOF MS analysis, the α-cyano-4-hydroxycinnamic acid (CHCA) matrix was used for the analysis (Shaaban et al. 2024). One µL of each sample solution (PEG: 1 mg/mL, ExoQuick: 330-fold dilution) was spotted onto the MALDI plate and air-dried. Subsequently, 1 µL of CHCA matrix solution (10 mg/mL, 2.5% trifluoroacetic acid (TFA) in 50% acetonitrile/water) was applied to the dried sample spot. MALDI-TOF MS analysis was performed using an ASTA MicroIDSys MALDI-TOF MS system (ASTA Inc., Suwon, Korea), equipped with a diode-pumped UV solid-state laser system. Mass spectra were acquired in positive reflection mode, with 300 laser shots collected per spectrum over an *m/z* scan range of 3000 to 50,000. Data analysis was conducted using IDSys Analysis software (Version 2).

### Scanning electron microscopy

Scanning electron microscopy (SEM; model AIS1800C, SERON Technologies Inc., Republic of Korea), (CNU Chemistry Core Facility, Daejeon, South Korea) was conducted to determine the size, morphology, and distribution of exosomes. For sample preparation before SEM analysis, 1 µL of the exosome sample was deposited onto a silicon wafer. The sample was fixed with 2.5 µL of 4% glutaraldehyde and dehydrated gradually using ethanol concentrations of 25%, 50%, 75%, 90%, and 100%, followed by an overnight incubation at 37 °C. Subsequently, the sample was coated with gold (Au) for 180 s in an ion coater (SPT-20, Elim Global, Gyeonggi-do, Korea). The SEM images were acquired at an accelerating voltage of 20.0 kV and a magnification of 34,000× for detailed examination.

### Nanoparticle tracking analysis (NTA)

NTA was conducted through the ZetaView PMX-430-Z QUATT laser system with a wavelength of 488 nm, and a fixed cell assembly for assessing average size distribution and particle concentration (Particle Metrix, Germany). The ZetaView v8.05.16 SP3 software was utilized for data analysis. Before measurement, samples were acclimated at room temperature (approximately 20 °C to 25 °C) for 30 to 45 min. To achieve the optimal particle concentration for the ZetaView system, sample dilutions ranged from 1:10,000 to 1:100,000 in PBS. All measurements were carried out under consistent conditions, including room temperature, pH 7.0, sensitivity set to 80, and shutter speed at 100 with eleven positions per replicate.

### Lysis of the enriched exosomes

Before Western blot analysis, the enriched exosome pellet (four-step PEG-based precipitation) was lysed by incubating it for 30 min at 4 °C in 2× radioimmunoprecipitation assay (RIPA) buffer (1:1 ratio). The RIPA buffer composition included 1.0% sodium deoxycholate, 2.0% NP-40, 100 mM Tris-HCl, 300 mM NaCl, 1 mM ethylenediaminetetraacetic acid (EDTA), 0.2% SDS, and protease inhibitors (cOmplete, EDTA-free Protease Inhibitor Cocktail Tablets, Roche). Subsequently, SDS-polyacrylamide gel electrophoresis (SDS-PAGE) experiments were conducted using in-house prepared tris-HCl and polyacrylamide gel (Jalaludin et al. 2023b).

### Western blot analysis

Western blotting analysis was conducted as previously described in previous reports with minor modifications (Park et al. 2023; Smolarz et al. 2019). For Western blot analysis via SDS-PAGE, each lysed exosome or supernatant sample (10 µL) was combined with 2.5 µL of 5× lane marker non-reducing sample buffer. The mixture was subjected to a 10-minute incubation at 60 °C, followed by centrifugation at 13,000 × g for 5 min. Lysed exosome proteins (20 µL each) were separated using the established gel electrophoresis method and transferred onto a PVDF membrane (Bio-Rad, catalog number: 162–0177). Next, the membrane was immersed in PBST blocking buffer containing 5% non-fat milk and 0.1% Tween 20 in PBS at room temperature for 1 h. Subsequently, it was incubated overnight at 4 °C with primary antibodies, including mouse anti-CD63, anti-CD9, rabbit anti-albumin, and Apolipoprotein antibodies. Following triplicate washes with PBST, the membrane was treated with secondary antibodies: preadsorbed goat anti-mouse IgG H&L (horseradish peroxidase) (Abcam catalog number: ab97040), diluted 1:2500 in PBST. Specific bands from each exosome sample were visualized by employing electrochemiluminescence (ECL) Western blotting detection reagent (Super Signal West Dura from Thermo Scientific, USA) on the membrane, and detection of the chemiluminescent signal was carried out using Kwik Quant Pro Imager (Kindle Biosciences, D1010, USA) according to the manufacturer’s instructions.

### Protein extraction and S-Trap digestion

Protein digestion was performed following the manufacturer’s instructions using an S-trap column. In brief, 500 µg of enriched exosome fractions were solubilized in 5% SDS in 50 mM TEAB, followed by vortexing to lyse the sample and dissolve proteins. The protein was reduced with 1 µL of 120 mM tris (2-carboxyethyl) phosphine (TCEP) (Thermo Scientific, USA) to the sample, followed by incubation at 55 °C for 15 min. The proteins were then alkylated by adding 2 µL of 1 M iodoacetamide and incubated at room temperature in the dark for 10 min. Subsequently, the alkylated proteins were acidified with phosphoric acid to a final concentration of 1.1%, and mixed with 175 µL of binding buffer (100 mM TEAB; 90% MeOH; pH 7.1). The protein solution was loaded onto S-Trap microcolumns (Protifi, USA) and centrifuged at 4000 × g for 30 s. The precipitated proteins were retained on the column, while impurities were washed away using several washes with triethylammonium bicarbonate (TEAB) in methanol. Proteolytic digestion was carried out by incubating the retained proteins with trypsin (Promega, Madison, WI) at 37 °C for 16 h at a protein-to-enzyme ratio of 10:1 (w/w). Peptides were then eluted sequentially using three elution buffers of increasing polarity (80 µL of 50 mM TEAB in water, 80 µL of 0.2% formic acid, and 80 µL of 50% acetonitrile). The resulting peptide mixtures were pooled, speed-vacuum dried, and finally reconstituted in 0.1% formic acid for nano-LC-MS/MS analysis.

### Nano-LC-MS/MS analysis

Following exosome enrichment using PEG precipitation and protein digestion, proteomic analysis was conducted using nano-LC-MS/MS. Chromatographic separation was achieved using a C18 column (Double nanoViper™ PepMap™ column, 75 μm x 500 mm, 2 μm, P/N DNV75500PN) on an RSLC nano U3000 system, interfaced with an Orbitrap Exploris 240 mass spectrometer. The mobile phases consisted of an aqueous solution containing 0.1% formic acid (solvent A) and acetonitrile containing 0.1% formic acid (solvent B). The sample was loaded at a flow rate of 400 nL/min, and peptides were separated at a flow rate of 300 nL/min. The gradient was 150 min in total, starting with 2% to 20% solvent B over 130 min. The gradient then continued to 32% over the next 20 min.

The nanospray source was operated with a spray voltage of 1.9 kV and a capillary temperature of 275 °C. Full MS scans were acquired with a resolution of 90,000, an automatic gain control (AGC) target of 3 × 10^6^ ions, and a maximum injection time of 80 ms, across an m/z range of 350 to 1200. For MS/MS (MS2) acquisition, the top 15 most intense ions were selected for fragmentation (TopN), with an AGC target of 1 × 10^6^ ions, a resolution of 30,000, and a maximum injection time of 200 ms. The isolation window was set to *m/z* 2, and fragmentation was carried out using higher-energy collisional dissociation (HCD) at a normalized collision energy of 30%. The MS2 spectra were recorded in profile mode, with a dynamic exclusion of 20 s and an intensity threshold of 1 × 10^5^ ions.

### Database search

The data files obtained for exosome analysis by nano-LC-MS/MS were processed by MaxQuant software version 2.5.0.0 and were searched against the human reference proteome sequence downloaded from Uniprot (Proteome ID: UP000005640; Organism ID: 9606; Reviewed (Swiss-Prot). The search parameters included an MS/MS ion search, utilizing trypsin as the proteolytic enzyme, with up to two missed cleavages permitted. Oxidation (M) and Acetyl (Protein N-term) were set as variable modifications, and Carbamidomethyl(C) was specified as a fixed modification. Peptide ions were identified with a mass accuracy tolerance of 5 ppm, and fragment ions were determined with a tolerance of 0.1 Da. All other parameters were set as default values, and the peptide identification results were filtered with a 1% false discovery rate (FDR) threshold.

## Results and discussion

### Exosome enrichment by PEG precipitation

In this study, PEG precipitation was systematically applied in the exosome isolation from serum samples. PEG precipitation has already been introduced for exosome enrichment, demonstrating simplicity, cost-effectiveness by eliminating the need for expensive commercial kits, and proven effectiveness and reproducibility (Ludwig et al. 2018; Rider et al. 2016; Stam et al. 2021; Weng et al. 2016). Along with exosomes, PEG has been utilized extensively for precipitating viral particles, nucleic acids, and various biomolecules (Gab-Allah et al., 2024; Gardiner et al. 2016; Zhou et al. 2020). The underlying mechanism of precipitation in the presence of PEG remains incompletely understood. The predominant theoretical frameworks explaining this phenomenon include the theory of excluded volume and the theory of attractive depletion forces (Lohmann et al., 2020). According to the excluded volume model, molecules precipitate due to reduced hydration and solubility in the presence of the polymer, and the theory of attractive depletion explains precipitation by the effect of attraction of molecules caused by the osmotic pressure of a PEG solution (Atha et al., 1981). The performance of PEG precipitation in isolating exosomes has been shown to depend on factors such as the molecular weight of PEG, its concentration during mixing with the sample, incubation temperature, and initial exosome concentration (Rider et al. 2016; Weng et al. 2016; Yakubovich et al., 2022).

**Fig. 2:**
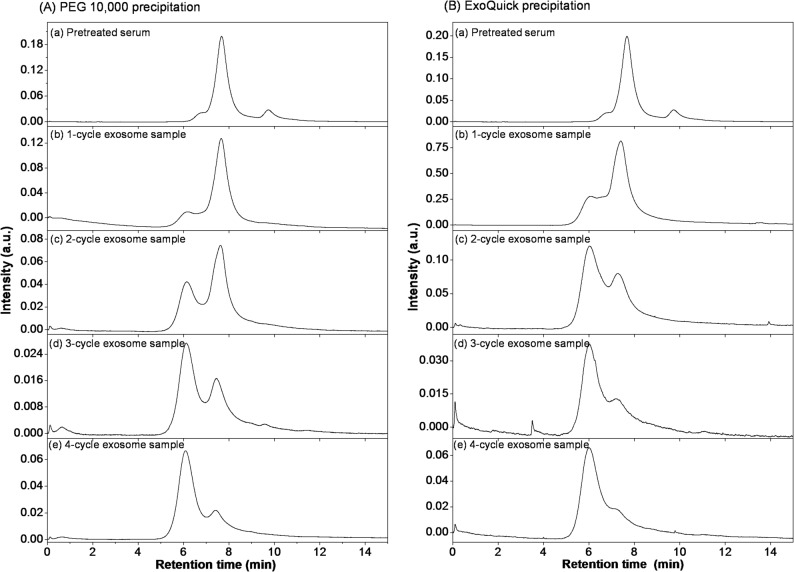
SEC chromatograms obtained with UV detection at 280 nm for pretreated serum and exosome samples enriched through 1-cycle, 2-cycle, 3-cycle, and 4-cycle steps using (**A**) PEG 10,000 and (**B**) ExoQuick. The percentage of PEG 10,000 used for the enrichment was 3.3 (w/v)%

During the initial development of this exosome enrichment method using PEG precipitation, size exclusion chromatography (SEC) was employed to assess the effectiveness of exosome isolation and enrichment from serum samples (Park et al. 2023). SEC operates on the principle of separating particles based on their size using a column filled with porous beads, thereby providing a clear profile of the sample composition before and after enrichment (Zhang et al. 2020). Large particles, such as extracellular vesicles (EVs), cannot penetrate the pores of these beads and elute from the column relatively quickly, while smaller particles, such as proteins, enter the pores and traverse a larger volume, resulting in a longer retention time (Jalaludin et al. 2021; Lai et al. 2022). The SEC results to evaluate the exosome isolation efficiency of PEG with varying molecular weights of 8,000, 10,000, 12,000, 20,000, and 35,000 Da, with a 3.3 (w/v)% PEG, as well as the commercial ExoQuick reagent, up to four cycles of enrichment are presented in Supplementary Figure S1. Figure 2 shows the SEC chromatograms using PEG 10,000 and ExoQuick for pretreated serum and exosome samples enriched through 1-cycle, 2-cycle, 3-cycle, and 4-cycle steps, while the corresponding UV contour spectra obtained with PDA detection were provided in Supplementary Figure S2.

The SEC analysis of pretreated serum samples without any enrichment revealed a single prominent peak, at around 8 min in Fig. 2, corresponding to serum proteins, indicating the absence of distinct exosome populations. As the samples were subjected to successive cycles of enrichment, either by PEG or ExoQuick, notable changes in the chromatograms were observed. The SEC chromatograms of the 2-cycle exosome samples displayed two distinct peaks: the first eluted peak at a retention time of around 6 min representing exosomes, and the second eluted peak at a retention time of around 8 min corresponding to residual proteins. This separation clearly demonstrated the initial success in isolating exosomes from the serum. With each additional cycle of enrichment, the relative intensity of the exosome peak increased, while the intensity of the protein peak gradually decreased. By the fourth cycle, the exosome peak was markedly more prominent, and the protein peak was significantly diminished, confirming the efficacy of the enrichment process.

As shown in Fig. 2, ExoQuick, being a commercial polymer-based reagent optimized for rapid exosome aggregation, exhibits slightly greater exosome peaks in the first three cycles, whereas PEG appears marginally higher at cycle 4. This subtle variation could be attributed to differences in precipitation or recovery behavior. ExoQuick’s polymer-based formulation, which may also contain salts, surfactants, or proprietary enhancers, can initially recover a larger fraction of readily precipitable exosomes, while PEG typically requires successive cycles to achieve comparable efficiency. Minor variations in viscosity, residual proteins, or exosome aggregation could also contribute to this effect. To the best of our knowledge, this is the first study to evaluate multiple enrichment cycles using PEG while directly comparing its efficiency with the widely used commercial kit “ExoQuick”. Although ExoQuick exhibits slightly higher peaks in cycles 1–3, the overall trends across all four cycles consistently demonstrate that PEG and ExoQuick provide comparable exosome enrichment, supporting PEG as a reliable and cost-effective alternative to the commercial kit.

Notably, a slight shift toward shorter retention times was also observed for the later-eluting protein peak (at a retention time of ~ 8 min) across successive enrichment cycles (Fig. 2). This behavior likely results from two combined effects: (i) progressive changes in serum protein composition as specific proteins are partially depleted during enrichment, and (ii) minor matrix effects arising from residual PEG or ExoQuick components, which can alter sample viscosity and modulate SEC column interactions. These factors may collectively influence the elution behavior of the remaining protein species. Importantly, these small shifts do not affect the identification of the exosome peak and are consistent with the physicochemical changes expected during repeated enrichment. Supplementary Figure S3 shows the SEC chromatograms of bovine serum albumin and human immunoglobulin G, whose peaks appeared at 8.0 and 7.3 min, respectively, confirming that the later-eluting peak around 8 min originates from residual proteins inherently present in the original serum samples.

The efficiency of PEG precipitation can also be influenced by PEG molecular weight, as it affects the polymer’s ability to induce exosome aggregation and sedimentation. Supplementary Figure S4 shows the overlap of SEC chromatograms for the exosome enriched through a 4-cycle step using PEG 8,000, 10,000, 12,000, 20,000, 35,000, and ExoQuick. In the fourth cycle analysis, the exosome peak was normalized to 100% across all samples to maintain consistency in evaluating the protein contamination profile. This approach allowed direct comparison by focusing only on the variability of the protein peak intensity, a critical indicator of the purity of the isolated exosomes. Our findings indicated that the highest protein peak intensity was observed with PEG 8,000 and PEG 10,000, followed by PEG 12,000, ExoQuick, PEG 20,000, and PEG 35,000, in descending order. Given that lower protein peak intensity correlates with improved exosome purity, these results initially suggested that PEG 35,000 exhibited superior performance in reducing protein contamination. However, larger PEGs such as PEG 35,000, PEG 20,000, and PEG 12,000 at a concentration of 3.3 (w/v)% have an issue with their solubility, requiring prolonged sonication time and generating their own precipitation. In addition, the 33 (w/v)% PEG 35,000 solution was too viscous to measure the exact volume for pipetting. MALDI-TOF MS analysis of the ExoQuick reagent and all the PEG solutions (Supplementary Figure S5) revealed that the molecular weight of the ExoQuick solution closely aligned with PEG 10,000, suggesting a compositional similarity between both reagents. Based on these findings, PEG 10,000 was selected for direct comparison with ExoQuick. This selection allowed for a robust assessment of PEG 10,000 as a potential cost-effective and efficient alternative to ExoQuick for exosome isolation, which is in line with other previous investigations using PEG 10,000 (García-Romero et al. 2019; Weng et al. 2016).

### Effect of PEG concentrations on exosome enrichment

To investigate the effect of PEG concentration on PEG-based serum exosome enrichment, exosome isolation efficiencies were evaluated using different PEG concentrations of 1.7, 3.3, 6.6, 11.0, and 16.5 (w/v)%. The results of each PEG concentration up to the four enrichment cycles are shown in Supplementary Figure S6, while the results from the fourth cycle across all concentrations are provided in Supplementary Figure S7. While higher PEG concentrations resulted in larger exosome pellets, these conditions did not yield optimal results for exosome isolation and purification based on Supplementary Figure S7. This is likely due to PEG’s potential to reduce the solubility of both exosomes and proteins, resulting in their co-precipitation and decreased exosome purity (Balaji et al., 2023; Kim et al. l^2020^). Conversely, lower PEG concentrations were also associated with reduced exosome recovery, possibly due to insufficient PEG concentration for effective exosome precipitation. Among the tested concentrations, the PEG concentration of 3.3% achieved the best balance between exosome yield and purity, producing the highest exosome peak intensity in SEC measurements. At this ratio, the precipitated pellets were clearly visible to the naked eye, confirming the successful isolation of exosome-enriched fractions from serum samples.

### Scanning electron microscopy (SEM)

**Fig. 3:**
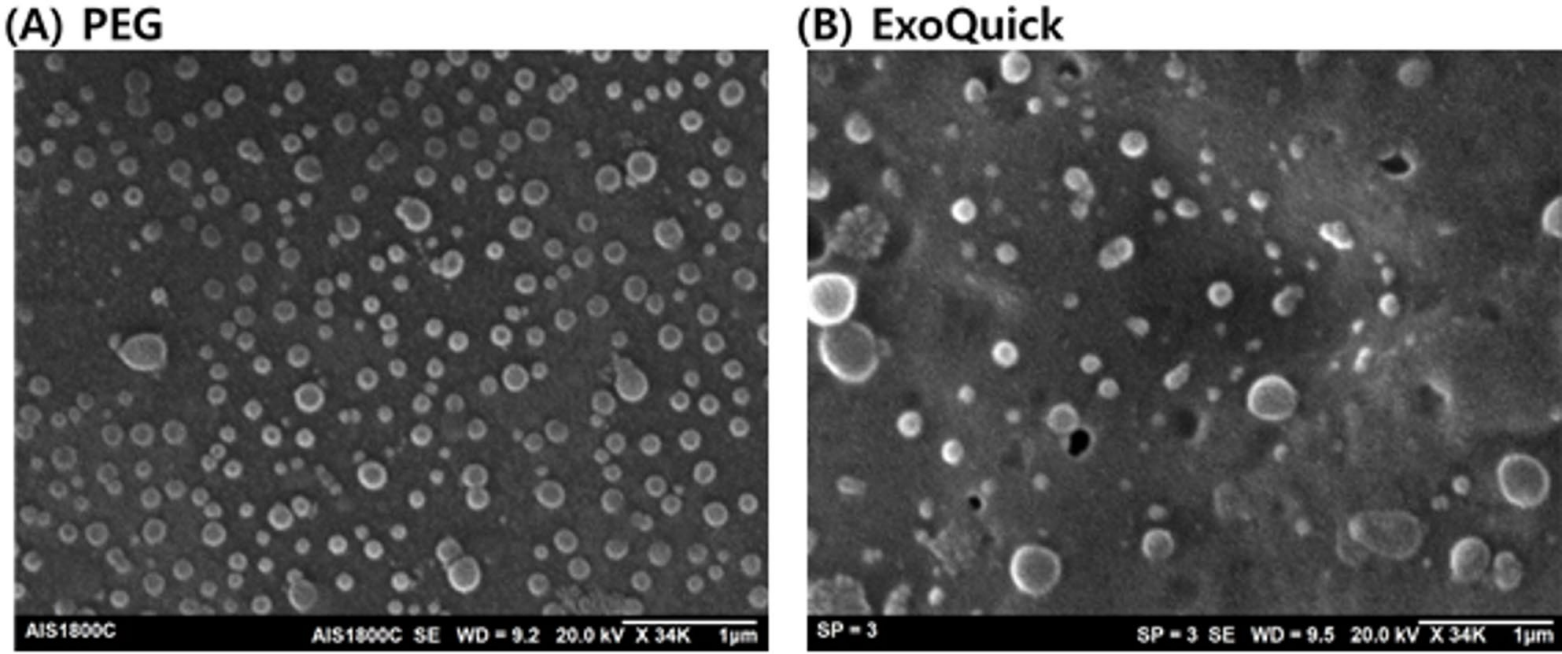
SEM images of 4-cycle exosome samples obtained from four cycles of exosome precipitation using (**A**) PEG 10,000 and (**B**) ExoQuick techniques

In this study, SEM was employed to analyze the morphology and size distribution of isolated exosomes. As shown in Fig. 3, SEM images of samples following 4-cycle PEG-precipitation isolated by PEG and ExoQuick revealed spherical vesicles characteristic of exosomes, with sizes ranging from 82 to 181 nm in diameter for both precipitation techniques. These dimensions are consistent with typical exosome sizes reported in the literature (El Ouahabi et al. 2021; Park et al. 2023; Weng et al. 2016). The SEM images further confirmed the integrity and intact morphology of the isolated exosomes, demonstrating the effectiveness of our isolation method in preserving vesicle structure. This size range is indicative of a pure exosome population, as larger particles and debris were effectively excluded during the isolation process. These findings underscore the suitability of our approach for obtaining high-quality exosome samples suitable for further detailed characterization and functional studies in both research and clinical applications.

### Western blot analysis

Western blotting is commonly used in research to detect and identify specific proteins. This method involves separating a protein mixture by molecular weight using SDS polyacrylamide gel electrophoresis, followed by binding with specific antibodies (Osborne et al., 2006). In the current investigation, Western blot analysis was performed to evaluate the efficacy of exosome enrichment using both PEG and ExoQuick precipitation methods. This study focused on detecting major exosomal markers (CD63 and CD9) and non-specific exosomal proteins (albumin and apolipoprotein) to evaluate the purity of the isolated exosomes.

**Fig. 4:**
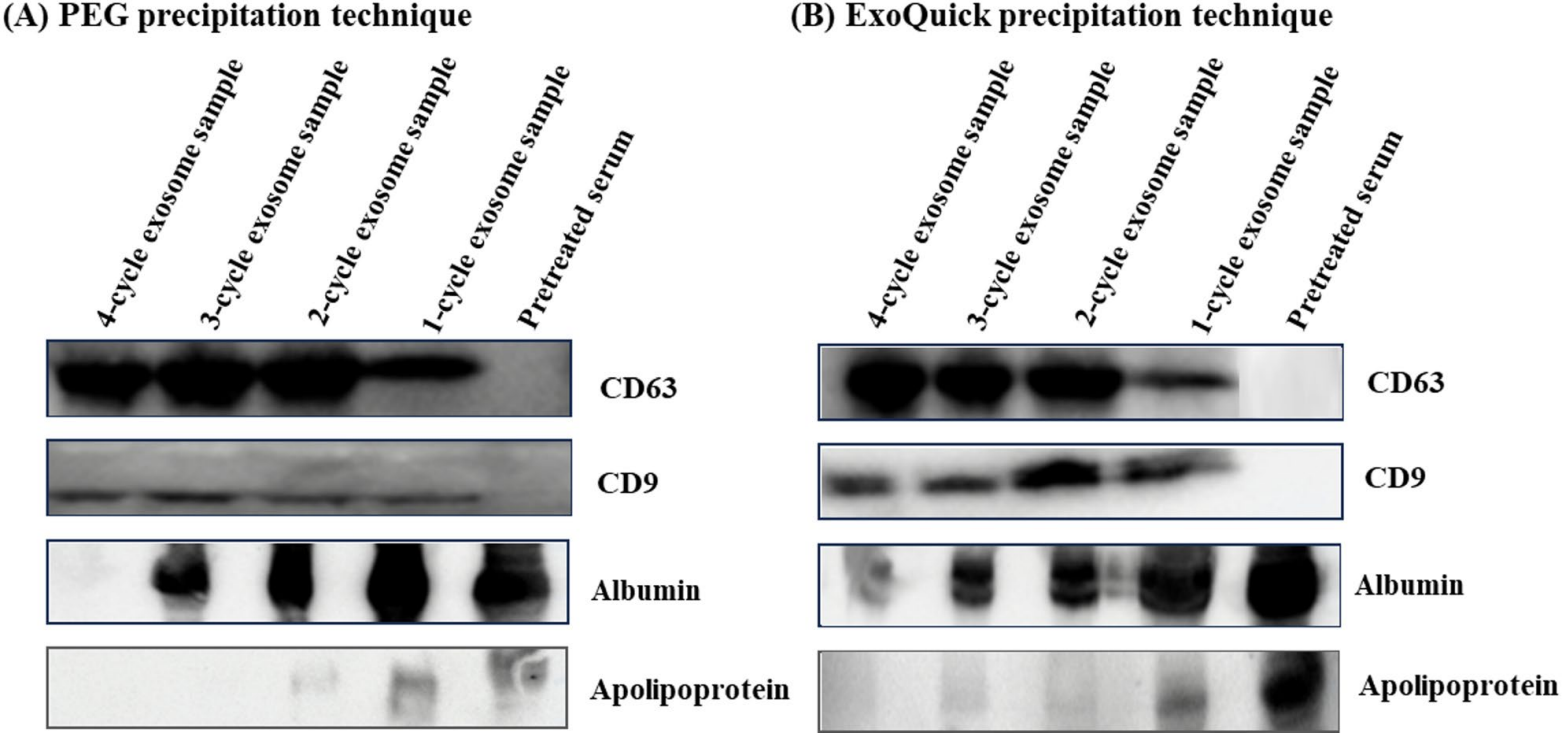
Western blot analysis results of detecting exosomal markers (CD63 and CD9) and non-exosomal proteins (albumin and apolipoprotein) present in pretreated serum, 1-cycle, 2-cycle, 3-cycle, and 4-cycle exosome-enriched samples using (**A**) PEG and (**B**) ExoQuick precipitation techniques

Figure 4 presents the results of Western blot analysis for these exosomal markers and proteins across different enrichment cycles using both PEG and ExoQuick precipitation methods. For the samples enriched using PEG precipitation, Western blot results demonstrated a progressive increase in the band intensity of CD63 and CD9 with each PEG enrichment cycle. This increase indicates a successful enrichment of exosomes, as these markers are specific to exosomal membranes. In contrast, the band intensity for albumin and apolipoprotein, which are not typically abundant in exosomes, showed a marked decrease with each enrichment step. Notably, by the third and fourth enrichment cycles, bands for apolipoprotein were no longer detectable, and albumin was absent by the fourth cycle (see Fig. 4A). This suggests that the PEG method effectively removes non-specific exosomal proteins, resulting in a highly purified exosome preparation.

For the samples obtained with the ExoQuick kit, there was a noticeable increase in CD63 and CD9 band intensity with each enrichment cycle, similar to the PEG precipitation method. However, even after multiple cycles of enrichment, minor band intensities for albumin and apolipoprotein were still present in the third and fourth-cycle exosome samples (see Fig. 4B). This indicates that while ExoQuick is effective in enriching exosomes from biological fluids, some non-specific exosomal proteins were retained. This behavior may result from ExoQuick’s proprietary formulation, which may include additional components, such as salts, surfactants, or other precipitation enhancers that promote co-precipitation of other abundant serum proteins. In contrast, PEG enrichment over successive cycles appears to reduce non-specific protein retention, consistent with the observed trends. This highlights PEG precipitation as a highly effective technique for exosome isolation.

Overall, the comparison between the two methods revealed that PEG precipitation yielded higher purity exosome preparations compared to the ExoQuick kit. The disappearance of band intensities for albumin and apolipoprotein in PEG-enriched samples (4-cycle exosome fraction) underscores its superior efficiency in isolating exosomes, making it a more reliable and cost-effective method for exosome enrichment in various applications. These findings highlight the potential of PEG precipitation as an alternative to more expensive commercial kits, offering both high yield and purity in exosome isolation.

### Nanoparticle tracking analysis (NTA)

**Fig. 5:**
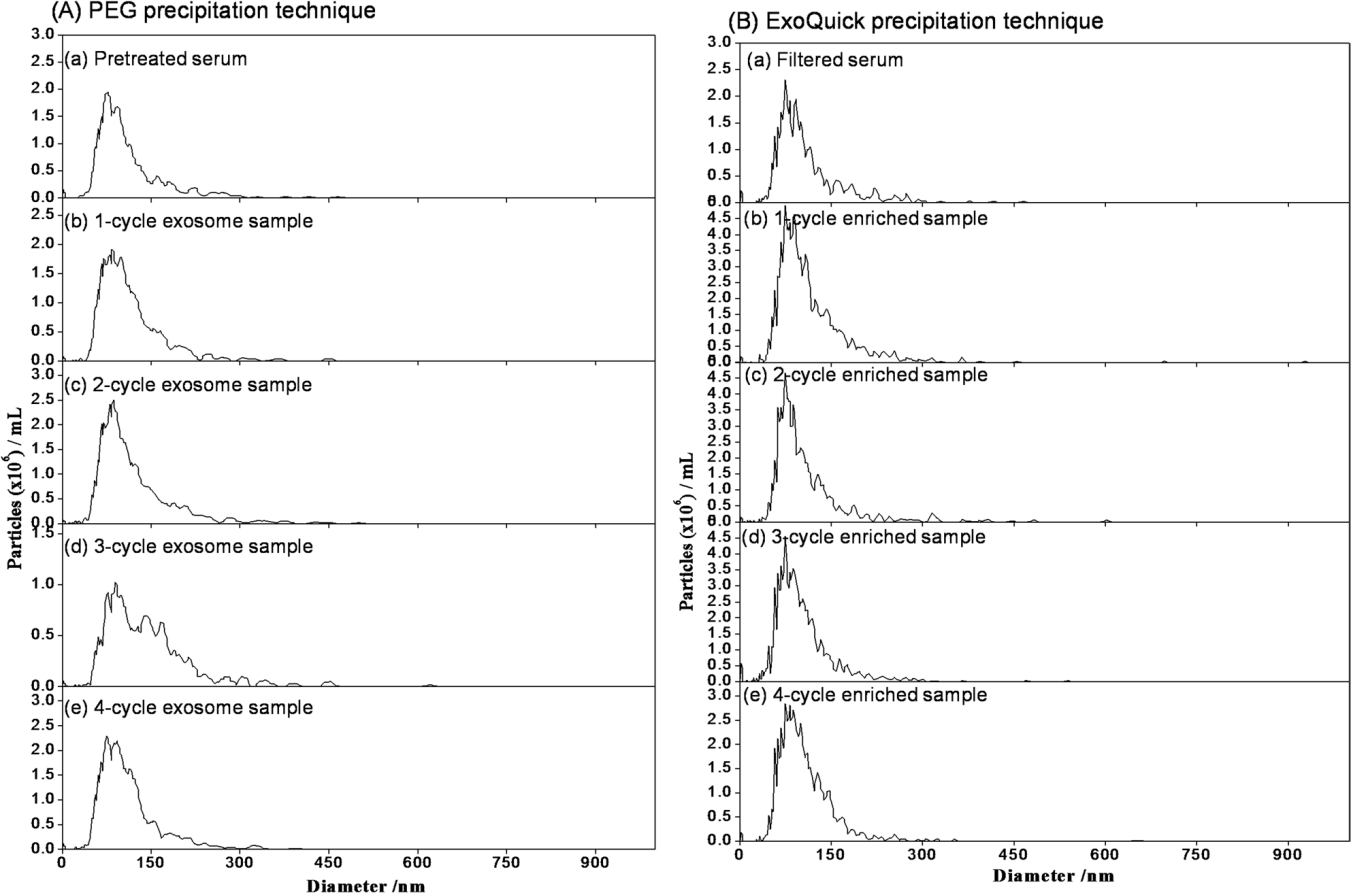
Graphical representation of size distribution curves determined by nanoparticle tracking analysis (NTA) measurements for pretreated serum, 1-cycle, 2-cycle, 3-cycle, and 4-cycle exosome samples obtained using (**A**) PEG and (**B**) ExoQuick precipitation techniques

NTA is a powerful analytical technique that is often used to characterize exosomes, including their size, concentration, and dynamics in a liquid suspension (Andreu et al. 2016; Ding et al. 2018; Macías et al. 2019; Willms et al. 2016). The particle size distributions of 4-cycle exosome samples from PEG and ExoQuick, obtained using NTA, are displayed in Fig. 5 and summarized in Table 1. The results indicate that the PEG precipitation technique and the ExoQuick kit yielded different profiles in terms of exosome concentration and size across the four enrichment cycles. For the PEG technique, there was an initial increase in concentration from the serum baseline (2.9 × 10¹¹ particles/mL) to the first (3.5 × 10¹² particles/mL) and second cycles (3.5 × 10¹² particles/mL). While the concentration slightly decreased in the third (1.3 × 10¹² particles/mL) and fourth cycles (1.2 × 10¹² particles/mL), the average exosome size increased from 119.7 nm to 138.7 nm, indicating a progressive enrichment of larger exosomes and removal of smaller particles or impurities. This suggests that the PEG technique is particularly effective at isolating larger exosomes, which are often of significant biological interest (Willms et al. 2016).

**Table 1:** NTA results summarizing the particle concentration, average size, median size, and standard deviation in pretreated serum, 1-cycle, 2-cycle, 3-cycle, and 4-cycle exosome samples following PEG and exoquick precipitation techniques

Sample	Enrichment technique	Parameters			
		Concentration (particles/mL)	Average size (nm)	Median size (nm)	Standard deviation (nm)
Pretreated serum	PEG	2.9 × 10^11^	120.6	110.3	46.7
	ExoQuick	3.4 × 10^11^	84.8	100.2	49.2
1-cycle exosome sample	PEG	3.5 × 10^12^	119.7	110.9	42.3
	ExoQuick	8.1 × 10^12^	105.3	89.0	54.5
2-cycle exosome sample	PEG	3.5 × 10^12^	117.9	109.7	46.2
	ExoQuick	6.3 × 10^12^	99.2	81.8	55.3
3-cycle exosome sample	PEG	1.3 × 10^12^	131.1	126.0	54.6
	ExoQuick	6.8 × 10^12^	94.3	82.3	44.4
4-cycle exosome sample	PEG	1.2 × 10^12^	138.7	126.7	59.6
	ExoQuick	4.9 × 10^12^	98.8	87.3	42.5

On the other hand, the ExoQuick technique consistently yielded higher exosome concentrations across the cycles, starting from 8.1 × 10¹² particles/mL in the first cycle and gradually decreasing to 4.9 × 10¹² particles/mL by the fourth cycle. The average size of exosomes also showed slight fluctuations, starting at 105.3 nm in the first cycle, with a decrease to 94.3 nm in the third cycle, and a slight increase to 98.8 nm in the fourth cycle. These observations suggest that the ExoQuick kit retains a higher concentration of smaller-sized exosomes throughout the cycles, whereas the PEG method demonstrates a unique advantage in selectively enriching larger exosomes over successive cycles, highlighting its effectiveness as a cost-efficient and facile alternative for targeted exosome isolation.

### Nano-LC-MS/MS profiling of exosome proteins

**Fig. 6:**
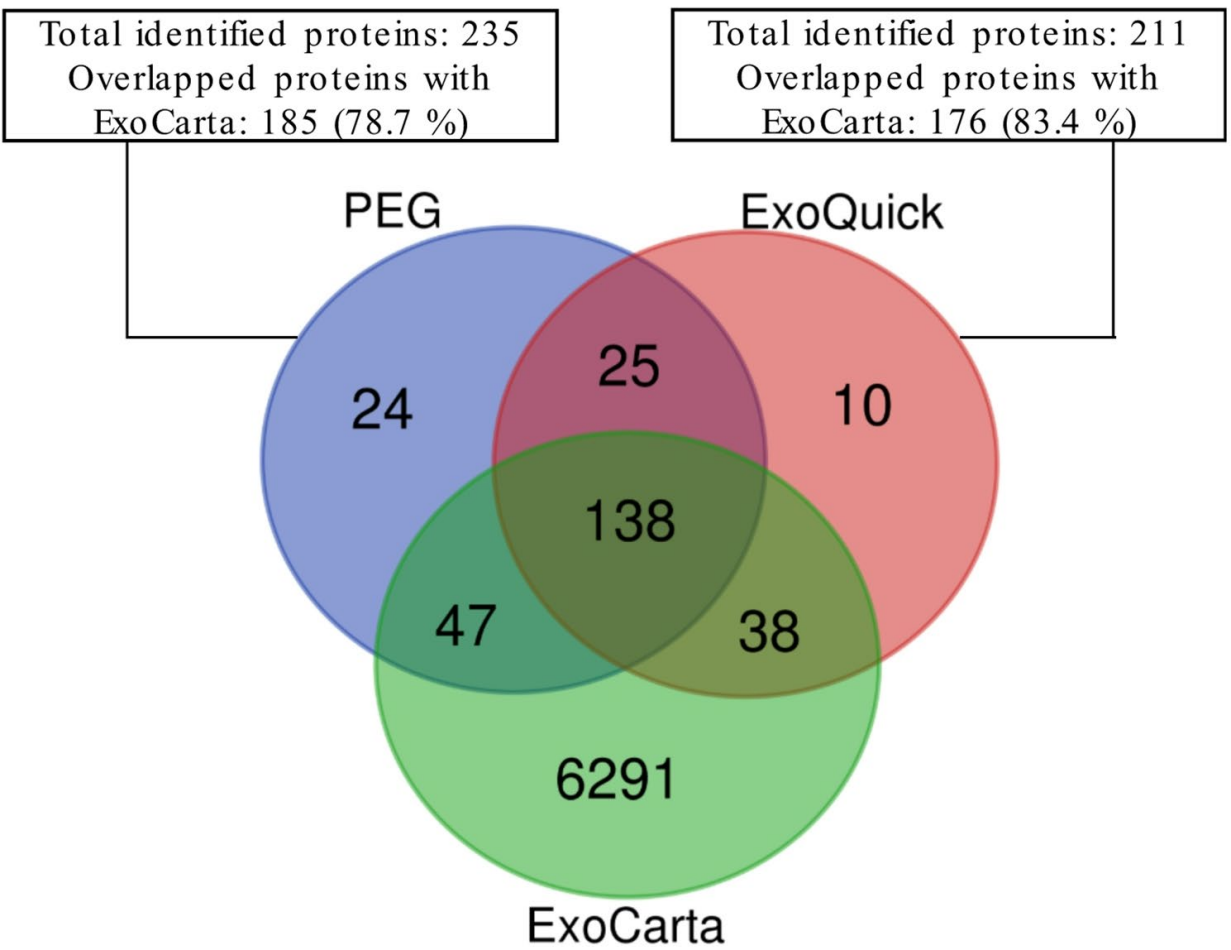
Venn diagram showing the overlap of proteins identified from exosomes enriched using the PEG and ExoQuick techniques, compared to the ExoCarta database

Nano-LC-MS/MS analyses of the 4-cycle exosome samples enriched using PEG 10,000 and ExoQuick techniques revealed a comprehensive profile of proteins present in exosome-enriched samples, with 235 proteins identified from exosomes isolated using the PEG precipitation technique and 211 proteins from those enriched with ExoQuick kits. The nano-LC-MS/MS chromatograms of exosome-enriched samples isolated using both techniques are depicted in Supplementary Figure S8, while the complete list of exosome proteins identified following the PEG precipitation technique and the ExoQuick kits can be found in Supplementary Table S1 and Table S2, respectively. To validate the identified proteins, they were compared against the available exosome protein database (ExoCarta, http://www.exocarta.org), which catalogs known exosomal proteins. A Venn diagram comparing the identified exosomal proteins from the PEG and ExoQuick enrichment techniques with exosomal proteins from the ExoCarta database is presented in Fig. 6. According to the ExoCarta database, among the 235 proteins identified using the PEG precipitation technique, 185 proteins (78.7%) were found to be exosomal proteins. Similarly, among the 211 proteins identified using the ExoQuick precipitation technique, 176 proteins (83.4%) were identified as exosomal proteins. A total of 148 exosomal proteins were commonly identified across the three groups: the PEG and ExoQuick precipatation techniques and the ExoCarta database (Table 2).

**Table 2:** A list of 138 exosomal proteins commonly identified from the exosome samples enriched by PEG and exoquick and from the exocarta database

No	Protein IDs	Protein names	Gene names
1	P60709	Actin, cytoplasmic 1	ACTB
2	P01011	Alpha-1-antichymotrypsin	SERPINA3
3	P01009	Alpha-1-antitrypsin	SERPINA1
4	P08697	Alpha-2-antiplasmin	SERPINF2
5	P02765	Alpha-2-HS-glycoprotein	AHSG
6	P01023	Alpha-2-macroglobulin	A2M
7	P01019	Angiotensinogen	AGT
8	P07355	Annexin A2	ANXA2
9	P01008	Antithrombin-III	SERPINC1
10	P02647	Apolipoprotein A-I	APOA1
11	P02652	Apolipoprotein A-II	APOA2
12	P06727	Apolipoprotein A-IV	APOA4
13	C0JYY2	Apolipoprotein B-100	APOB
14	P02656	Apolipoprotein C-III	APOC3
15	A5YAK2	Apolipoprotein C-IV	APOC4
16	C9JF17	Apolipoprotein D	APOD
17	P02649	Apolipoprotein E	APOE
18	O95445	Apolipoprotein M	APOM
19	P08519	Apolipoprotein(a)	LPA
20	P02749	Beta-2-glycoprotein 1	APOH
21	P04003	C4b-binding protein alpha chain	C4BPA
22	P20851	C4b-binding protein beta chain	C4BPB
23	G3XAP6	Cartilage oligomeric matrix protein	COMP
24	P04040	Catalase	CAT
25	P49913	Cathelicidin antimicrobial peptide	CAMP
26	O43866	CD5 antigen-like	CD5L
27	P21926	CD9 antigen	CD9
28	B7Z5Q2	Ceruloplasmin	CP
29	Q9BY43	Charged multivesicular body protein 4a	CHMP4A
30	P11597	Cholesteryl ester transfer protein	CETP
31	P10909	Clusterin	CLU
32	P12259	Coagulation factor V	F5
33	P00488	Coagulation factor XIII A chain	F13A1
34	Q9BWP8	Collectin-11	COLEC11
35	P02745	Complement C1q subcomponent subunit A	C1QA
36	P02746	Complement C1q subcomponent subunit B	C1QB
37	A0A3B3ISR2	Complement C1r subcomponent	C1R
38	P09871	Complement C1s subcomponent	C1S
39	P01024	Complement C3	C3
40	P0C0L4	Complement C4-A	C4A
41	P0C0L5	Complement C4-B	C4B
42	P01031	Complement C5	C5
43	P10643	Complement component C7	C7
44	P07357	Complement component C8 alpha chain	C8A
45	P07360	Complement component C8 gamma chain	C8G
46	P02748	Complement component C9	C9
47	P81605	Dermcidin	DCD
48	Q08554	Desmocollin-1	DSC1
49	Q02413	Desmoglein-1	DSG1
50	P15924	Desmoplakin	DSP
51	Q16610	Extracellular matrix protein 1	ECM1
52	Q01469	Fatty acid-binding protein, epidermal	FABP5
53	P02671	Fibrinogen alpha chain	FGA
54	P02675	Fibrinogen beta chain	FGB
55	P02679	Fibrinogen gamma chain	FGG
56	P23142	Fibulin-1	FBLN1
57	Q15485	Ficolin-2	FCN2
58	O75636	Ficolin-3	FCN3
59	Q5D862	Filaggrin-2	FLG2
60	P21333	Filamin-A	FLNA
61	Q08380	Galectin-3-binding protein	LGALS3BP
62	P06396	Gelsolin	GSN
63	P04406	Glyceraldehyde-3-phosphate dehydrogenase	GAPDH
64	P00738	Haptoglobin	HP
65	P00739	Haptoglobin-related protein	HPR
66	P69905	Hemoglobin subunit alpha	HBA1
67	P68871	Hemoglobin subunit beta	HBB
68	P02790	Hemopexin	HPX
69	P20671	Histone H2A type 1-D	HIST1H2AD
70	P68431	Histone H3.1	HIST1H3A
71	P62805	Histone H4	HIST1H4A
72	Q86YZ3	Hornerin	HRNR
73	A0A5H1ZRQ7	Ig lambda-7 chain C region	IGLC7
74	P01871	Ig mu chain C region	IGHM
75	Q9Y6R7	IgGFc-binding protein	FCGBP
76	P01591	Immunoglobulin J chain	IGJ
77	P08514	Integrin alpha-Iib	ITGA2B
78	P05106	Integrin beta-3	ITGB3
79	P19827	Inter-alpha-trypsin inhibitor heavy chain H1	ITIH1
80	P19823	Inter-alpha-trypsin inhibitor heavy chain H2	ITIH2
81	Q14624	Inter-alpha-trypsin inhibitor heavy chain H4	ITIH4
82	P14923	Junction plakoglobin	JUP
83	P29622	Kallistatin	SERPINA4
84	P13645	Keratin, type I cytoskeletal 10	KRT10
85	P02533	Keratin, type I cytoskeletal 14	KRT14
86	P08779	Keratin, type I cytoskeletal 16	KRT16
87	Q04695	Keratin, type I cytoskeletal 17	KRT17
88	P35527	Keratin, type I cytoskeletal 9	KRT9
89	P35908	Keratin, type II cytoskeletal 2 epidermal	KRT2
90	P13647	Keratin, type II cytoskeletal 5	KRT5
91	B4DRR0	Keratin, type II cytoskeletal 6 A	KRT6A
92	Q8N1N4	Keratin, type II cytoskeletal 78	KRT78
93	Q5T749	Keratinocyte proline-rich protein	KPRP
94	P01042	Kininogen-1	KNG1
95	P18428	Lipopolysaccharide-binding protein	LBP
96	P48740	Mannan-binding lectin serine protease 1	MASP1
97	O00187	Mannan-binding lectin serine protease 2	MASP2
98	P11226	Mannose-binding protein C	MBL2
99	Q6P4Q7	Metal transporter CNNM4	CNNM4
100	P35579	Myosin-9	MYH9
101	Q5T2W1	Na(+)/H(+) exchange regulatory cofactor NHE-RF3	PDZK1
102	O76041	Nebulette	NEBL
103	P59665	Neutrophil defensin 1	DEFA1
104	P62937	Peptidyl-prolyl cis-trans isomerase A	PPIA
105	P05155	Plasma protease C1 inhibitor	SERPING1
106	P05154	Plasma serine protease inhibitor	SERPINA5
107	P00747	Plasminogen	PLG
108	P02776	Platelet factor 4	PF4
109	P13224	Platelet glycoprotein Ib beta chain	GP1BB
110	P01833	Polymeric immunoglobulin receptor	PIGR
111	P0CG8	Polyubiquitin-C	UBC
112	Q9UHG3	Prenylcysteine oxidase 1	PCYOX1
113	P07737	Profilin-1	PFN1
114	P02760	Protein AMBP	AMBP
115	P05109	Protein S100-A8	S100A8
116	P06702	Protein S100-A9	S100A9
117	Q8TF72	Protein Shroom3	SHROOM3
118	Q92954	Proteoglycan 4	PRG4
119	P00734	Prothrombin	F2
120	P14618	Pyruvate kinase PKM	PKM
121	P61224	Ras-related protein Rap-1b	RAP1B
122	Q13103	Secreted phosphoprotein 24	SPP2
123	P49908	Selenoprotein P	SEPP1
124	P02787	Serotransferrin	TF
125	P02768	Serum albumin	ALB
126	P0DJI8	Serum amyloid A-1 protein	SAA1
127	P02743	Serum amyloid P-component	APCS
128	P27169	Serum paraoxonase/arylesterase 1	PON1
129	Q9Y490	Talin-1	TLN1
130	P24821	Tenascin	TNC
131	P10599	Thioredoxin	TXN
132	P07996	Thrombospondin-1	THBS1
133	P35443	Thrombospondin-4	THBS4
134	P02766	Transthyretin	TTR
135	P35030	Trypsin-3	PRSS3
136	P68363	Tubulin alpha-1B chain	TUBA1B
137	P02774	Vitamin D-binding protein	GC
138	P07225	Vitamin K-dependent protein S	PROS1

These shared proteins illustrate that both PEG and ExoQuick enrichment techniques are effective and emphasize the successful isolation of exosomes, with PEG providing slightly broader coverage. Among the common exosomal proteins identified in both PEG and ExoQuick preparations are CD9, Apolipoprotein A-I, Annexin A2, glyceraldehyde-3-phosphate dehydrogenase (GAPDH), and actin. These proteins are widely recognized as being involved in exosome biogenesis and fusion with target cells, cellular communication, lipid metabolism, and glycolysis (Burke et al. 2014; Doyle et al., 2019; Wu et al. 2023). Additionally, platelet factor 4 (PF4), a novel exosome marker protein (Nguyen et al. 2019), was identified in both preparation methods. In addition to these markers, other commonly exosomal proteins identified in PEG samples included heat shock protein 70 (HSP70), alpha-enolase, and alpha-actinin-1. These proteins further support the exosomal origin of the samples, as they are involved in cargo sorting, structural integrity, and vesicular trafficking (Doyle et al., 2019). Additionally, proteins such as integrins and alpha-actinin-4, which are also frequently found in exosomes, were identified, contributing to the vesicle’s role in cell adhesion and signal transduction.

The comparable protein profiles between the PEG method and the ExoQuick kit highlight the efficiency of the PEG technique as a cost-effective alternative for exosome enrichment. Despite its lower cost and simplicity, the PEG method demonstrated similar proteomic coverage, capturing a broad spectrum of exosomal proteins without significant loss of key markers or functional proteins.

## Conclusions

A PEG precipitation method using 3.3 (w/v)% PEG 10,000 was successfully established for exosome enrichment from human serum, and its performance was compared with that of the commercially available ExoQuick kit. It was observed that the PEG precipitation method, implemented up to four enrichment cycles, demonstrated high efficacy in exosome isolation, as confirmed by particle concentration and size distribution metrics using NTA. Additionally, SEC verified the progressive reduction of protein contaminants with each enrichment cycle and the increasing prominence of the exosome peak, highlighting the method’s effectiveness in purifying exosomes. Western blotting further validated the presence of key exosomal markers, including CD63 and CD9, in the enriched samples. Moreover, SEM analysis revealed well-preserved exosomes within the expected size range (81–181 nm), with no obvious structural deformation. Moreover, proteomic profiling via nano-LC-MS/MS identified 185 and 176 previously reported exosomal proteins from PEG and ExoQuick samples, respectively (based on the ExoCarta database), thereby underscoring the technique’s capacity to maintain exosome integrity and protein content throughout the enrichment process. Importantly, this approach offers a cost-effective, straightforward, and scalable solution, requiring no sophisticated instrumentation, making it particularly advantageous for large-scale or resource-constrained laboratories. In comparison to the more expensive ExoQuick kit, PEG precipitation yielded comparable exosome quality, further demonstrating its utility as a viable alternative. As exosome-based research and clinical applications continue to expand, the demand for scalable, affordable, and efficient isolation techniques will increase. The PEG precipitation technique described in this study holds significant potential to address these needs, offering a practical solution for routine exosome isolation across diverse research and clinical environments. This PEG-based approach can be readily adopted in clinical or diagnostic laboratories for cost-efficient exosome isolation.

## Supplementary Information

Below is the link to the electronic supplementary material.

Supplementary Material 1

## Data Availability

The supporting data are included in the electronic supplementary material.
