# Supplementary material for: Exosome enrichment from human serum using polyethylene glycol precipitation
Source: J Anal Sci Technol. 2025 Dec 23;16(1):46. doi: 10.1186/s40543-025-00521-0 (PMC12727789; doi:10.1186/s40543-025-00521-0)
Supplement: Supplementary file 1 — Supplementary Material 1 [file 40543_2025_521_MOESM1_ESM.docx]

**Exosome enrichment from human serum using polyethylene glycol precipitation**

**Table S1.** List of 235 proteins identified from exosomes isolated using the PEG 10,000 precipitation technique.

| No. | Protein IDs | Protein names | Gene names | Unique peptides | Sequence coverage [%] | Mol. weight [kDa] |
| --- | --- | --- | --- | --- | --- | --- |
| 1 | P31947 | 14-3-3 protein sigma | SFN | 1 | 12.9 | 23.379 |
| 2 | P63104 | 14-3-3 protein zeta/delta | YWHAZ | 2 | 20.8 | 65.865 |
| 3 | P62280 | 40S ribosomal protein S11 | RPS11 | 1 | 5.1 | 575.89 |
| 4 | P62273 | 40S ribosomal protein S29 | RPS29 | 1 | 19.6 | 21.717 |
| 5 | P62701 | 40S ribosomal protein S4, X isoform | RPS4X | 1 | 4.9 | 82.42 |
| 6 | P68133 | Actin, alpha skeletal muscle | ACTA1 | 1 | 26.5 | 36.053 |
| 7 | P60709 | Actin, cytoplasmic 1 | ACTB | 5 | 43.7 | 15.998 |
| 8 | Q9HDC9 | Adipocyte plasma membrane-associated protein | APMAP | 2 | 5.5 | 56.865 |
| 9 | B2R773 | Adiponectin | ADIPOQ | 1 | 6.1 | 55.154 |
| 10 | Q8TD30 | Alanine aminotransferase 2 | GPT2 | 1 | 1.9 | 53.321 |
| 11 | P19652 | Alpha-1-acid glycoprotein 2 | ORM2 | 7 | 36.3 | 192.78 |
| 12 | P01011 | Alpha-1-antichymotrypsin | SERPINA3 | 5 | 13.9 | 10.845 |
| 13 | P01009 | Alpha-1-antitrypsin | SERPINA1 | 1 | 42.6 | 216.85 |
| 14 | P08697 | Alpha-2-antiplasmin | SERPINF2 | 8 | 22.2 | 51.676 |
| 15 | P02765 | Alpha-2-HS-glycoprotein | AHSG | 2 | 6.5 | 70.036 |
| 16 | P01023 | Alpha-2-macroglobulin | A2M | 46 | 40.2 | 7.695 |
| 17 | O43707 | Alpha-actinin-4 | ACTN4 | 1 | 1.6 | 104.85 |
| 18 | P06733 | Alpha-enolase | ENO1 | 1 | 2.8 | 47.168 |
| 19 | P01019 | Angiotensinogen | AGT | 9 | 21 | 11.648 |
| 20 | P07355 | Annexin A2 | ANXA2 | 2 | 6.2 | 38.604 |
| 21 | P01008 | Antithrombin-III | SERPINC1 | 20 | 46.6 | 22.11 |
| 22 | P02647 | Apolipoprotein A-I | APOA1 | 23 | 68.5 | 30.777 |
| 23 | P02652 | Apolipoprotein A-II | APOA2 | 5 | 41 | 32.903 |
| 24 | P06727 | Apolipoprotein A-IV | APOA4 | 6 | 44.2 | 94.972 |
| 25 | C0JYY2 | Apolipoprotein B-100 | APOB | 132 | 60.5 | 70.154 |
| 26 | K7ERI9 | Apolipoprotein C-I | APOC1 | 5 | 37.7 | 15.164 |
| 27 | K7ER74 | Apolipoprotein C-II | APOC4-APOC2 | 4 | 33.1 | 54.305 |
| 28 | P02656 | Apolipoprotein C-III | APOC3 | 3 | 34.3 | 49.092 |
| 29 | A5YAK2 | Apolipoprotein C-IV | APOC4 | 2 | 22 | 10.529 |
| 30 | C9JF17 | Apolipoprotein D | APOD | 6 | 26.5 | 71.957 |
| 31 | P02649 | Apolipoprotein E | APOE | 2 | 74.4 | 11.118 |
| 32 | Q13790 | Apolipoprotein F | APOF | 1 | 4.3 | 70.108 |
| 33 | O95445 | Apolipoprotein M | APOM | 6 | 36.2 | 15.054 |
| 34 | P08519 | Apolipoprotein(a) | LPA | 1 | 49.5 | 272.32 |
| 35 | Q9Y2X7 | ARF GTPase-activating protein GIT1 | GIT1 | 1 | 2 | 11.805 |
| 36 | Q9UII2 | ATPase inhibitor, mitochondrial | ATPIF1 | 1 | 9.4 | 57.903 |
| 37 | O95817 | BAG family molecular chaperone regulator 3 | BAG3 | 1 | 3.3 | 12.903 |
| 38 | P02749 | Beta-2-glycoprotein 1 | APOH | 3 | 15.1 | 10.957 |
| 39 | P04003 | C4b-binding protein alpha chain | C4BPA | 2 | 67.3 | 83.283 |
| 40 | P20851 | C4b-binding protein beta chain | C4BPB | 10 | 51.2 | 192.75 |
| 41 | G3XAP6 | Cartilage oligomeric matrix protein | COMP | 7 | 18.5 | 64.419 |
| 42 | A9UFC0 | Caspase-14 | CASP14 | 1 | 4.5 | 90.568 |
| 43 | P04040 | Catalase | CAT | 2 | 5.3 | 10.856 |
| 44 | P49913 | Cathelicidin antimicrobial peptide | CAMP | 3 | 20 | 11.252 |
| 45 | O43866 | CD5 antigen-like | CD5L | 21 | 62 | 66.038 |
| 46 | P21926 | CD9 antigen | CD9 | 1 | 4.4 | 10.348 |
| 47 | Q15642 | Cdc42-interacting protein 4 | TRIP10 | 1 | 1.8 | 13.921 |
| 48 | B7Z5Q2 | Ceruloplasmin | CP | 13 | 17.8 | 12.897 |
| 49 | Q9BY43 | Charged multivesicular body protein 4a | CHMP4A | 1 | 3.2 | 52.049 |
| 50 | P11597 | Cholesteryl ester transfer protein | CETP | 1 | 1.6 | 57.129 |
| 51 | Q96N23 | Cilia- and flagella-associated protein 54 | CFAP54 | 1 | 0.4 | 41.272 |
| 52 | Q8N684 | Cleavage and polyadenylation specificity factor subunit 7 | CPSF7 | 1 | 3.4 | 59.512 |
| 53 | P10909 | Clusterin | CLU | 17 | 36.3 | 10.313 |
| 54 | P12259 | Coagulation factor V | F5 | 6 | 4.9 | 10.383 |
| 55 | P03951 | Coagulation factor XI | F11 | 1 | 1.1 | 20.825 |
| 56 | P00748 | Coagulation factor XII | F12 | 2 | 2.8 | 10.748 |
| 57 | P00488 | Coagulation factor XIII A chain | F13A1 | 21 | 30.5 | 41.736 |
| 58 | P05160 | Coagulation factor XIII B chain | F13B | 3 | 7.3 | 11.175 |
| 59 | A6NI56 | Coiled-coil domain-containing protein 154 | CCDC154 | 1 | 1.6 | 28.538 |
| 60 | Q5M9N0 | Coiled-coil domain-containing protein 158 | CCDC158 | 1 | 0.9 | 13.739 |
| 61 | O75534 | Cold shock domain-containing protein E1 | CSDE1 | 1 | 1.5 | 13.315 |
| 62 | Q9Y6Z7 | Collectin-10 | COLEC10 | 2 | 6.9 | 44.786 |
| 63 | Q9BWP8 | Collectin-11 | COLEC11 | 3 | 15.1 | 17.877 |
| 64 | P02745 | Complement C1q subcomponent subunit A | C1QA | 3 | 44.1 | 26.721 |
| 65 | P02746 | Complement C1q subcomponent subunit B | C1QB | 6 | 37.2 | 25.773 |
| 66 | P02747 | Complement C1q subcomponent subunit C | C1QC | 7 | 37.1 | 37.429 |
| 67 | A0A3B3ISR2 | Complement C1r subcomponent | C1R | 2 | 64 | 49.874 |
| 68 | P09871 | Complement C1s subcomponent | C1S | 28 | 54.5 | 67.033 |
| 69 | P01024 | Complement C3 | C3 | 29 | 75.4 | 298.38 |
| 70 | P0C0L4 | Complement C4-A | C4A | 1 | 57.7 | 309.26 |
| 71 | P0C0L5 | Complement C4-B | C4B | 1 | 57.7 | 10.834 |
| 72 | P01031 | Complement C5 | C5 | 71 | 48.4 | 57.936 |
| 73 | P10643 | Complement component C7 | C7 | 19 | 31.6 | 226.54 |
| 74 | P07357 | Complement component C8 alpha chain | C8A | 6 | 14.2 | 127.14 |
| 75 | P07358 | Complement component C8 beta chain | C8B | 14 | 22.5 | 61.594 |
| 76 | P07360 | Complement component C8 gamma chain | C8G | 7 | 48 | 55.928 |
| 77 | P02748 | Complement component C9 | C9 | 10 | 19 | 13.28 |
| 78 | Q03591 | Complement factor H-related protein 1 | CFHR1 | 2 | 54.8 | 53.5 |
| 79 | A0A8V8TQS1 | Complement factor H-related protein 2 | CFHR2 | 3 | 50 | 46.736 |
| 80 | Q92496 | Complement factor H-related protein 4 | CFHR4 | 2 | 5 | 25.702 |
| 81 | Q9BXR6 | Complement factor H-related protein 5 | CFHR5 | 17 | 34.6 | 104.72 |
| 82 | P02741 | C-reactive protein | CRP | 2 | 8.9 | 45.205 |
| 83 | P81605 | Dermcidin | DCD | 3 | 22.7 | 280.74 |
| 84 | Q08554 | Desmocollin-1 | DSC1 | 3 | 4.1 | 39.731 |
| 85 | Q02413 | Desmoglein-1 | DSG1 | 7 | 10.8 | 77.213 |
| 86 | P15924 | Desmoplakin | DSP | 31 | 11.7 | 54.565 |
| 87 | P53602 | Diphosphomevalonate decarboxylase | MVD | 1 | 2.2 | 23.602 |
| 88 | Q8TF46 | DIS3-like exonuclease 1 | DIS3L | 1 | 0.7 | 9.9272 |
| 89 | P49411 | Elongation factor Tu, mitochondrial | TUFM | 2 | 7 | 53.129 |
| 90 | Q16610 | Extracellular matrix protein 1 | ECM1 | 13 | 49.3 | 51.276 |
| 91 | P15311 | Ezrin | EZR | 1 | 2 | 80.198 |
| 92 | Q01469 | Fatty acid-binding protein, epidermal | FABP5 | 2 | 13.3 | 55.877 |
| 93 | P02671 | Fibrinogen alpha chain | FGA | 24 | 42.6 | 21.253 |
| 94 | P02675 | Fibrinogen beta chain | FGB | 15 | 42 | 83.267 |
| 95 | P02679 | Fibrinogen gamma chain | FGG | 11 | 29.4 | 75.122 |
| 96 | B4DUV1 | Fibulin-1 | FBLN1 | 4 | 34.6 | 50.151 |
| 97 | P23142 | Fibulin-1 | FBLN1 | 8 | 34.9 | 48.934 |
| 98 | Q15485 | Ficolin-2 | FCN2 | 2 | 7.3 | 10.201 |
| 99 | O75636 | Ficolin-3 | FCN3 | 6 | 19.7 | 11.737 |
| 100 | P20930 | Filaggrin | FLG | 2 | 2 | 18.331 |
| 101 | Q5D862 | Filaggrin-2 | FLG2 | 4 | 2.6 | 51.267 |
| 102 | P21333 | Filamin-A | FLNA | 4 | 2.6 | 13.546 |
| 103 | Q08380 | Galectin-3-binding protein | LGALS3BP | 11 | 23.2 | 13.304 |
| 104 | P47929 | Galectin-7 | LGALS7 | 5 | 43.4 | 26.143 |
| 105 | P06396 | Gelsolin | GSN | 12 | 20.5 | 10.687 |
| 106 | P04406 | Glyceraldehyde-3-phosphate dehydrogenase | GAPDH | 1 | 6.3 | 12.528 |
| 107 | P00738 | Haptoglobin | HP | 8 | 47 | 20.049 |
| 108 | P00739 | Haptoglobin-related protein | HPR | 7 | 56.6 | 8.647 |
| 109 | P17066 | Heat shock 70 kDa protein 6 | HSPA6 | 1 | 1.7 | 11.46 |
| 110 | P11142 | Heat shock cognate 71 kDa protein | HSPA8 | 1 | 1.5 | 6.6767 |
| 111 | P04792 | Heat shock protein beta-1 | HSPB1 | 1 | 7.8 | 11.895 |
| 112 | P69905 | Hemoglobin subunit alpha | HBA1 | 4 | 48.6 | 47.499 |
| 113 | P68871 | Hemoglobin subunit beta | HBB | 11 | 83 | 48.105 |
| 114 | P02790 | Hemopexin | HPX | 3 | 10.4 | 18.098 |
| 115 | P22626 | Heterogeneous nuclear ribonucleoproteins A2/B1 | HNRNPA2B1 | 1 | 4.8 | 46.708 |
| 116 | P20671 | Histone H2A type 1-D | HIST1H2AD | 3 | 26.9 | 15.257 |
| 117 | P68431 | Histone H3.1 | HIST1H3A | 2 | 10.3 | 36.638 |
| 118 | P62805 | Histone H4 | HIST1H4A | 4 | 38.8 | 50.077 |
| 119 | Q86YZ3 | Hornerin | HRNR | 16 | 21.8 | 435.16 |
| 120 | Q4G0P3 | Hydrocephalus-inducing protein homolog | HYDIN | 1 | 0.2 | 50.133 |
| 121 | P01880 | Ig delta chain C region | IGHD | 2 | 5.3 | 10.21 |
| 122 | Q6N093 | Ig gamma-2 chain C region | DKFZp686I04196 | 1 | 40.8 | 25.807 |
| 123 | P01860 | Ig gamma-3 chain C region | IGHG3 | 7 | 39.7 | 21.775 |
| 124 | A0A5C2GKQ6 | Ig heavy chain V-III region DOB | VH3 | 1 | 56.7 | 12.422 |
| 125 | A0A5C2GC73 | Ig kappa chain V-II region FR | IGKV2D-28 | 1 | 54.5 | 12.404 |
| 126 | Q5NV80 | Ig lambda chain V region 4A | V3-2 | 1 | 18.4 | 11.567 |
| 127 | A0M8Q7 | Ig lambda chain V-IV region Bau | V2-1 | 1 | 23.2 | 11.582 |
| 128 | A0A449C1A1 | Ig lambda chain V-VI region AR | VL6 | 1 | 55.1 | 239.62 |
| 129 | A0A5H1ZRQ7 | Ig lambda-7 chain C region | IGLC7 | 2 | 55.7 | 11.725 |
| 130 | P01871 | Ig mu chain C region | IGHM | 3 | 51.9 | 99.995 |
| 131 | Q9Y6R7 | IgGFc-binding protein | FCGBP | 6 | 2.5 | 10.384 |
| 132 | P01591 | Immunoglobulin J chain | IGJ | 10 | 72.3 | 18.431 |
| 133 | P08514 | Integrin alpha-Iib | ITGA2B | 8 | 9.5 | 25.387 |
| 134 | P05106 | Integrin beta-3 | ITGB3 | 3 | 4.4 | 11.506 |
| 135 | P19827 | Inter-alpha-trypsin inhibitor heavy chain H1 | ITIH1 | 5 | 8.6 | 45.674 |
| 136 | P19823 | Inter-alpha-trypsin inhibitor heavy chain H2 | ITIH2 | 15 | 16.5 | 240.85 |
| 137 | Q14624 | Inter-alpha-trypsin inhibitor heavy chain H4 | ITIH4 | 12 | 12.3 | 160.6 |
| 138 | O14896 | Interferon regulatory factor 6 | IRF6 | 1 | 3.9 | 37.154 |
| 139 | P48200 | Iron-responsive element-binding protein 2 | IREB2 | 1 | 0.7 | 331.77 |
| 140 | P14923 | Junction plakoglobin | JUP | 7 | 18.3 | 192.87 |
| 141 | P29622 | Kallistatin | SERPINA4 | 11 | 32.1 | 48.541 |
| 142 | P13645 | Keratin, type I cytoskeletal 10 | KRT10 | 30 | 59.1 | 13.228 |
| 143 | P02533 | Keratin, type I cytoskeletal 14 | KRT14 | 5 | 55.9 | 30.444 |
| 144 | P08779 | Keratin, type I cytoskeletal 16 | KRT16 | 15 | 59.8 | 25.098 |
| 145 | Q04695 | Keratin, type I cytoskeletal 17 | KRT17 | 8 | 39.8 | 24.409 |
| 146 | P35527 | Keratin, type I cytoskeletal 9 | KRT9 | 36 | 69.8 | 93.517 |
| 147 | P35908 | Keratin, type II cytoskeletal 2 epidermal | KRT2 | 1 | 82.6 | 515.55 |
| 148 | P13647 | Keratin, type II cytoskeletal 5 | KRT5 | 2 | 45.1 | 105.87 |
| 149 | B4DRR0 | Keratin, type II cytoskeletal 6A | KRT6A | 2 | 55 | 284.54 |
| 150 | Q8N1N4 | Keratin, type II cytoskeletal 78 | KRT78 | 3 | 7.3 | 68.124 |
| 151 | Q5T749 | Keratinocyte proline-rich protein | KPRP | 1 | 1.6 | 12.801 |
| 152 | O15066 | Kinesin-like protein KIF3B | KIF3B | 1 | 1.2 | 46.48 |
| 153 | P01042 | Kininogen-1 | KNG1 | 7 | 14.4 | 151.06 |
| 154 | Q14847 | LIM and SH3 domain protein 1 | LASP1 | 1 | 13 | 10.852 |
| 155 | P18428 | Lipopolysaccharide-binding protein | LBP | 5 | 12.5 | 76.684 |
| 156 | P07195 | L-lactate dehydrogenase B chain | LDHB | 1 | 4.5 | 70.897 |
| 157 | P48740 | Mannan-binding lectin serine protease 1 | MASP1 | 8 | 36.2 | 53.383 |
| 158 | O00187 | Mannan-binding lectin serine protease 2 | MASP2 | 7 | 11.7 | 88.884 |
| 159 | P11226 | Mannose-binding protein C | MBL2 | 4 | 16.5 | 139.09 |
| 160 | O60244 | Mediator of RNA polymerase II transcription subunit 14 | MED14 | 1 | 0.6 | 47.251 |
| 161 | Q6P4Q7 | Metal transporter CNNM4 | CNNM4 | 1 | 0.9 | 19.91 |
| 162 | O75431 | Metaxin-2 | MTX2 | 1 | 3.8 | 27.666 |
| 163 | O43684 | Mitotic checkpoint protein BUB3 | BUB3 | 1 | 8.5 | 81.744 |
| 164 | Q8NCY6 | Myb/SANT-like DNA-binding domain-containing protein 4 | MSANTD4 | 1 | 2.9 | 248.07 |
| 165 | P35579 | Myosin-9 | MYH9 | 3 | 1.8 | 13.299 |
| 166 | Q5T2W1 | Na(+)/H(+) exchange regulatory cofactor NHE-RF3 | PDZK1 | 1 | 3.1 | 49.67 |
| 167 | O76041 | Nebulette | NEBL | 1 | 0.8 | 68.351 |
| 168 | P59665 | Neutrophil defensin 1 | DEFA1 | 2 | 19.1 | 28.357 |
| 169 | Q5SRE5 | Nucleoporin NUP188 homolog | NUP188 | 1 | 0.4 | 5.6854 |
| 170 | P62937 | Peptidyl-prolyl cis-trans isomerase A | PPIA | 1 | 5.5 | 11.799 |
| 171 | Q06830 | Peroxiredoxin-1 | PRDX1 | 2 | 8.5 | 10.539 |
| 172 | A0A0A0MS09 | Peroxiredoxin-4 | PRDX4 | 2 | 8.5 | 10.539 |
| 173 | Q13835 | Plakophilin-1 | PKP1 | 2 | 3.5 | 43.404 |
| 174 | P05155 | Plasma protease C1 inhibitor | SERPING1 | 15 | 28 | 77.038 |
| 175 | P05154 | Plasma serine protease inhibitor | SERPINA5 | 2 | 5.4 | 14.808 |
| 176 | P00747 | Plasminogen | PLG | 29 | 47.5 | 24.654 |
| 177 | Q8NC51 | Plasminogen activator inhibitor 1 RNA-binding protein | SERBP1 | 1 | 3.9 | 226.51 |
| 178 | P02776 | Platelet factor 4 | PF4 | 3 | 35.6 | 13.512 |
| 179 | P13224 | Platelet glycoprotein Ib beta chain | GP1BB | 2 | 9.2 | 57.838 |
| 180 | Q13093 | Platelet-activating factor acetylhydrolase | PLA2G7 | 3 | 8.6 | 29.597 |
| 181 | P01833 | Polymeric immunoglobulin receptor | PIGR | 8 | 11.1 | 85.124 |
| 182 | P0CG48 | Polyubiquitin-C | UBC | 2 | 32.8 | 11.422 |
| 183 | Q5VXH5 | PRAME family member 7 | PRAMEF7 | 1 | 3.2 | 244.4 |
| 184 | Q9UHG3 | Prenylcysteine oxidase 1 | PCYOX1 | 8 | 22.4 | 25.024 |
| 185 | Q14147 | Probable ATP-dependent RNA helicase DHX34 | DHX34 | 1 | 0.7 | 27.745 |
| 186 | P07737 | Profilin-1 | PFN1 | 1 | 11.4 | 15.525 |
| 187 | P27918 | Properdin | CFP | 17 | 35.6 | 11.142 |
| 188 | P02760 | Protein AMBP | AMBP | 4 | 14.5 | 58.794 |
| 189 | Q8WVV4 | Protein POF1B | POF1B | 1 | 1.7 | 52.851 |
| 190 | P05109 | Protein S100-A8 | S100A8 | 1 | 11.8 | 51.923 |
| 191 | P06702 | Protein S100-A9 | S100A9 | 2 | 24.6 | 29.717 |
| 192 | Q8TF72 | Protein Shroom3 | SHROOM3 | 1 | 0.5 | 13.096 |
| 193 | B4DQ50 | Protein-glutamine gamma-glutamyltransferase E | TGM3 | 2 | 3.9 | 35.399 |
| 194 | Q92954 | Proteoglycan 4 | PRG4 | 1 | 1.2 | 43.974 |
| 195 | P00734 | Prothrombin | F2 | 10 | 28.1 | 69.412 |
| 196 | O75061 | Putative tyrosine-protein phosphatase auxilin | DNAJC6 | 1 | 1.3 | 128.12 |
| 197 | P14618 | Pyruvate kinase PKM | PKM | 1 | 3 | 13.594 |
| 198 | Q92565 | Rap guanine nucleotide exchange factor 5 | RAPGEF5 | 1 | 2.6 | 52.602 |
| 199 | P61224 | Ras-related protein Rap-1b | RAP1B | 2 | 12.5 | 25.137 |
| 200 | P38159 | RNA-binding motif protein, X chromosome | RBMX | 1 | 3.6 | 13.242 |
| 201 | Q13103 | Secreted phosphoprotein 24 | SPP2 | 2 | 11.4 | 105.06 |
| 202 | P49908 | Selenoprotein P | SEPP1 | 2 | 6 | 14.107 |
| 203 | P02787 | Serotransferrin | TF | 19 | 32.7 | 187.15 |
| 204 | P02768 | Serum albumin | ALB | 44 | 69.8 | 101.39 |
| 205 | B2R5G8 | Serum amyloid A protein |  | 1 | 33.8 | 42.051 |
| 206 | P0DJI8 | Serum amyloid A-1 protein | SAA1 | 2 | 30.3 | 75.51 |
| 207 | A0A096LPE2 | Serum amyloid A-4 protein | SAA2-SAA4 | 1 | 25.5 | 11.238 |
| 208 | P02743 | Serum amyloid P-component | APCS | 6 | 27.4 | 39.029 |
| 209 | P27169 | Serum paraoxonase/arylesterase 1 | PON1 | 10 | 39.7 | 63.485 |
| 210 | I1VSB5 | Sialic acid-binding Ig-like lectin 16 | SIGLEC16 | 1 | 1.6 | 24.969 |
| 211 | Q695T7 | Sodium-dependent neutral amino acid transporter B(0)AT1 | SLC6A19 | 1 | 2.2 | 11.367 |
| 212 | Q13813 | Spectrin alpha chain, non-erythrocytic 1 | SPTAN1 | 1 | 0.5 | 25.416 |
| 213 | P26368 | Splicing factor U2AF 65 kDa subunit | U2AF2 | 1 | 6.9 | 49.328 |
| 214 | Q6UWP8 | Suprabasin | SBSN | 2 | 15.3 | 54.159 |
| 215 | Q9Y490 | Talin-1 | TLN1 | 5 | 3.4 | 196.71 |
| 216 | P24821 | Tenascin | TNC | 1 | 10.4 | 226.53 |
| 217 | P10599 | Thioredoxin | TXN | 2 | 21 | 52.917 |
| 218 | P07996 | Thrombospondin-1 | THBS1 | 28 | 34.5 | 25.038 |
| 219 | P35443 | Thrombospondin-4 | THBS4 | 13 | 22.1 | 79.696 |
| 220 | Q9Y2W1 | Thyroid hormone receptor-associated protein 3 | THRAP3 | 1 | 1.5 | 85.696 |
| 221 | P05549 | Transcription factor AP-2-alpha | TFAP2A | 1 | 1.8 | 11.714 |
| 222 | P02766 | Transthyretin | TTR | 8 | 68.7 | 10.162 |
| 223 | P07477 | Trypsin-1 | PRSS1 | 1 | 8.1 | 251.7 |
| 224 | P35030 | Trypsin-3 | PRSS3 | 1 | 4.3 | 12.75 |
| 225 | P68363 | Tubulin alpha-1B chain | TUBA1B | 4 | 14 | 116.45 |
| 226 | P07437 | Tubulin beta chain | TUBB | 1 | 2.3 | 26.016 |
| 227 | Q8IYN6 | UBA-like domain-containing protein 2 | UBALD2 | 1 | 5.5 | 25.021 |
| 228 | Q9BWL3 | Uncharacterized protein C1orf43 | C1orf43 | 1 | 3.2 | 12.469 |
| 229 | Q9BY89 | Uncharacterized protein KIAA1671 | KIAA1671 | 1 | 1 | 24.792 |
| 230 | P02774 | Vitamin D-binding protein | GC | 3 | 7.8 | 65.432 |
| 231 | P07225 | Vitamin K-dependent protein S | PROS1 | 22 | 43 | 26.558 |
| 232 | P04004 | Vitronectin | VTN | 16 | 39.3 | 26.59 |
| 233 | P04275 | von Willebrand factor | VWF | 4 | 27.9 | 43.831 |
| 234 | O75083 | WD repeat-containing protein 1 | WDR1 | 1 | 4.6 | 10.508 |
| 235 | P25311 | Zinc-alpha-2-glycoprotein | AZGP1 | 1 | 3.4 | 67.732 |

**Table S2.** List of 211 proteins identified from exosomes isolated using the ExoQuick precipitation technique.

| No. | Protein IDs | Protein names | Gene names | Unique peptides | Sequence coverage [%] | Mol. weight [kDa] |
| --- | --- | --- | --- | --- | --- | --- |
| 1 | P63104 | 14-3-3 protein zeta/delta | YWHAZ | 8 | 51.4 | 27.745 |
| 2 | P68032 | Actin, alpha cardiac muscle 1 | ACTC1 | 1 | 29.2 | 42.019 |
| 3 | P60709 | Actin, cytoplasmic 1 | ACTB | 1 | 49.1 | 41.736 |
| 4 | P63261 | Actin, cytoplasmic 2 | ACTG1 | 1 | 49.1 | 41.792 |
| 5 | B2R773 | Adiponectin | ADIPOQ | 1 | 6.1 | 26.425 |
| 6 | P02763 | Alpha-1-acid glycoprotein 1 | ORM1 | 1 | 12.4 | 23.539 |
| 7 | P19652 | Alpha-1-acid glycoprotein 2 | ORM2 | 2 | 20.9 | 23.602 |
| 8 | P01011 | Alpha-1-antichymotrypsin | SERPINA3 | 3 | 6.9 | 47.65 |
| 9 | P01009 | Alpha-1-antitrypsin | SERPINA1 | 1 | 45.2 | 46.736 |
| 10 | P08697 | Alpha-2-antiplasmin | SERPINF2 | 7 | 15.5 | 54.565 |
| 11 | P02765 | Alpha-2-HS-glycoprotein | AHSG | 3 | 8.4 | 39.34 |
| 12 | P01023 | Alpha-2-macroglobulin | A2M | 50 | 45.3 | 163.29 |
| 13 | P01019 | Angiotensinogen | AGT | 6 | 16 | 52.069 |
| 14 | P07355 | Annexin A2 | ANXA2 | 2 | 6.2 | 38.604 |
| 15 | P01008 | Antithrombin-III | SERPINC1 | 12 | 35.3 | 52.602 |
| 16 | P02647 | Apolipoprotein A-I | APOA1 | 25 | 72.7 | 30.777 |
| 17 | P02652 | Apolipoprotein A-II | APOA2 | 5 | 41 | 11.175 |
| 18 | P06727 | Apolipoprotein A-IV | APOA4 | 6 | 38.4 | 45.371 |
| 19 | Q6Q788 | Apolipoprotein A-V | APOA5 | 2 | 5.5 | 41.212 |
| 20 | C0JYY2 | Apolipoprotein B-100 | APOB | 125 | 60.4 | 515.55 |
| 21 | K7ERI9 | Apolipoprotein C-I | APOC1 | 5 | 37.7 | 8.647 |
| 22 | K7ER74 | Apolipoprotein C-II | APOC4-APOC2 | 4 | 33.1 | 20.049 |
| 23 | P02656 | Apolipoprotein C-III | APOC3 | 3 | 34.3 | 10.852 |
| 24 | P55056 | Apolipoprotein C-IV | APOC4 | 3 | 29.1 | 14.553 |
| 25 | C9JF17 | Apolipoprotein D | APOD | 6 | 26.5 | 24.158 |
| 26 | P02649 | Apolipoprotein E | APOE | 8 | 72.9 | 36.154 |
| 27 | O14791 | Apolipoprotein L1 | APOL1 | 2 | 42.2 | 43.974 |
| 28 | O95445 | Apolipoprotein M | APOM | 7 | 39.9 | 21.253 |
| 29 | Q1HP67 | Apolipoprotein(a) | LPA | 40 | 46.3 | 226.51 |
| 30 | Q6PL18 | ATPase family AAA domain-containing protein 2 | ATAD2 | 1 | 1.3 | 158.55 |
| 31 | Q9UII2 | ATPase inhibitor, mitochondrial | ATPIF1 | 1 | 9.4 | 12.249 |
| 32 | Q08211 | ATP-dependent RNA helicase A | DHX9 | 1 | 0.7 | 140.96 |
| 33 | P02730 | Band 3 anion transport protein | SLC4A1 | 6 | 8.2 | 101.79 |
| 34 | P02749 | Beta-2-glycoprotein 1 | APOH | 2 | 8.4 | 38.298 |
| 35 | P04003 | C4b-binding protein alpha chain | C4BPA | 2 | 63.1 | 67.033 |
| 36 | P20851 | C4b-binding protein beta chain | C4BPB | 10 | 51.2 | 28.357 |
| 37 | P04040 | Catalase | CAT | 3 | 6.3 | 59.755 |
| 38 | P49913 | Cathelicidin antimicrobial peptide | CAMP | 5 | 24.7 | 19.301 |
| 39 | O43866 | CD5 antigen-like | CD5L | 20 | 61.4 | 38.087 |
| 40 | P21926 | CD9 antigen | CD9 | 1 | 4.4 | 25.416 |
| 41 | B7Z5Q2 | Ceruloplasmin | CP | 9 | 11.6 | 108.82 |
| 42 | Q9BY43 | Charged multivesicular body protein 4a | CHMP4A | 1 | 3.2 | 25.098 |
| 43 | P11597 | Cholesteryl ester transfer protein | CETP | 1 | 1.6 | 54.756 |
| 44 | Q00610 | Clathrin heavy chain 1 | CLTC | 1 | 0.4 | 191.61 |
| 45 | P10909 | Clusterin | CLU | 17 | 35 | 52.494 |
| 46 | P12259 | Coagulation factor V | F5 | 3 | 3.1 | 251.7 |
| 47 | P00748 | Coagulation factor XII | F12 | 1 | 1.3 | 67.791 |
| 48 | P00488 | Coagulation factor XIII A chain | F13A1 | 18 | 27.9 | 83.267 |
| 49 | P05160 | Coagulation factor XIII B chain | F13B | 1 | 1.4 | 75.51 |
| 50 | P23528 | Cofilin-1 | CFL1 | 2 | 13.9 | 18.502 |
| 51 | A6NI56 | Coiled-coil domain-containing protein 154 | CCDC154 | 1 | 1.6 | 75.407 |
| 52 | P12111 | Collagen alpha-3(VI) chain | COL6A3 | 2 | 0.6 | 343.67 |
| 53 | Q9BWP8 | Collectin-11 | COLEC11 | 1 | 6.3 | 28.665 |
| 54 | P02745 | Complement C1q subcomponent subunit A | C1QA | 10 | 43.7 | 26.016 |
| 55 | P02746 | Complement C1q subcomponent subunit B | C1QB | 8 | 35.2 | 26.721 |
| 56 | P02747 | Complement C1q subcomponent subunit C | C1QC | 6 | 36.3 | 25.773 |
| 57 | A0A3B3ISR2 | Complement C1r subcomponent | C1R | 1 | 57 | 80.173 |
| 58 | P09871 | Complement C1s subcomponent | C1S | 25 | 47.8 | 76.684 |
| 59 | P01024 | Complement C3 | C3 | 93 | 67.2 | 187.15 |
| 60 | P0C0L4 | Complement C4-A | C4A | 1 | 53.8 | 192.78 |
| 61 | P0C0L5 | Complement C4-B | C4B | 1 | 53.8 | 192.75 |
| 62 | P01031 | Complement C5 | C5 | 66 | 45 | 188.3 |
| 63 | P13671 | Complement component C6 | C6 | 13 | 14.8 | 104.79 |
| 64 | P10643 | Complement component C7 | C7 | 13 | 20.9 | 93.517 |
| 65 | P07357 | Complement component C8 alpha chain | C8A | 9 | 17.5 | 65.163 |
| 66 | P07358 | Complement component C8 beta chain | C8B | 8 | 16.2 | 66.947 |
| 67 | P07360 | Complement component C8 gamma chain | C8G | 4 | 29.2 | 22.277 |
| 68 | P02748 | Complement component C9 | C9 | 10 | 20.4 | 63.173 |
| 69 | P00751 | Complement factor B | CFB | 2 | 3.3 | 85.532 |
| 70 | P08603 | Complement factor H | CFH | 2 | 46 | 139.09 |
| 71 | Q03591 | Complement factor H-related protein 1 | CFHR1 | 2 | 30.9 | 37.65 |
| 72 | A0A8V8TQS1 | Complement factor H-related protein 2 | CFHR2 | 3 | 22.4 | 28.538 |
| 73 | Q92496 | Complement factor H-related protein 4 | CFHR4 | 1 | 3.8 | 65.35 |
| 74 | Q9BXR6 | Complement factor H-related protein 5 | CFHR5 | 17 | 33.4 | 64.419 |
| 75 | P05156 | Complement factor I | CFI | 6 | 37.7 | 65.75 |
| 76 | P02741 | C-reactive protein | CRP | 2 | 8.9 | 25.038 |
| 77 | P81605 | Dermcidin | DCD | 2 | 20 | 11.284 |
| 78 | Q08554 | Desmocollin-1 | DSC1 | 2 | 3 | 99.986 |
| 79 | Q02413 | Desmoglein-1 | DSG1 | 3 | 3.8 | 113.75 |
| 80 | P15924 | Desmoplakin | DSP | 13 | 4.3 | 331.77 |
| 81 | P27105 | Erythrocyte band 7 integral membrane protein | STOM | 3 | 10.4 | 31.73 |
| 82 | Q16610 | Extracellular matrix protein 1 | ECM1 | 12 | 30.2 | 60.673 |
| 83 | Q01469 | Fatty acid-binding protein, epidermal | FABP5 | 1 | 6.7 | 15.164 |
| 84 | Q86UX7 | Fermitin family homolog 3 | FERMT3 | 5 | 8.4 | 75.952 |
| 85 | P02671 | Fibrinogen alpha chain | FGA | 25 | 39 | 94.972 |
| 86 | P02675 | Fibrinogen beta chain | FGB | 16 | 44.4 | 55.928 |
| 87 | P02679 | Fibrinogen gamma chain | FGG | 11 | 28.9 | 51.511 |
| 88 | B4DUV1 | Fibulin-1 | FBLN1 | 1 | 26.8 | 70.154 |
| 89 | Q15485 | Ficolin-2 | FCN2 | 3 | 10.2 | 34.001 |
| 90 | O75636 | Ficolin-3 | FCN3 | 7 | 24.7 | 32.903 |
| 91 | P20930 | Filaggrin | FLG | 1 | 0.2 | 435.16 |
| 92 | Q5D862 | Filaggrin-2 | FLG2 | 5 | 5.6 | 248.07 |
| 93 | P21333 | Filamin-A | FLNA | 34 | 16.4 | 280.74 |
| 94 | P04075 | Fructose-bisphosphate aldolase A | ALDOA | 2 | 6 | 39.42 |
| 95 | Q08380 | Galectin-3-binding protein | LGALS3BP | 11 | 18.1 | 65.33 |
| 96 | B7Z2X4 | Gelsolin | GSN | 12 | 19.9 | 77.788 |
| 97 | P04406 | Glyceraldehyde-3-phosphate dehydrogenase | GAPDH | 2 | 7.5 | 36.053 |
| 98 | P62879 | Guanine nucleotide-binding protein G(I)/G(S)/G(T) subunit beta-2 | GNB2 | 1 | 2.9 | 37.331 |
| 99 | P00738 | Haptoglobin | HP | 8 | 41.9 | 45.205 |
| 100 | P00739 | Haptoglobin-related protein | HPR | 7 | 59.2 | 39.029 |
| 101 | P69905 | Hemoglobin subunit alpha | HBA1 | 3 | 28.2 | 15.257 |
| 102 | D9YZU5 | Hemoglobin subunit beta | HBB | 2 | 76.9 | 15.998 |
| 103 | P02042 | Hemoglobin subunit delta | HBD | 1 | 49 | 16.055 |
| 104 | P02790 | Hemopexin | HPX | 5 | 13.4 | 51.676 |
| 105 | P05546 | Heparin cofactor 2 | SERPIND1 | 3 | 5 | 57.07 |
| 106 | B2R8I2 | Histidine-rich glycoprotein | HRG | 12 | 26.9 | 59.512 |
| 107 | P20671 | Histone H2A type 1-D | HIST1H2AD | 2 | 21.5 | 14.107 |
| 108 | Q99879 | Histone H2B type 1-M | HIST1H2BM | 2 | 13.5 | 13.989 |
| 109 | P68431 | Histone H3.1 | HIST1H3A | 1 | 5.1 | 15.404 |
| 110 | P62805 | Histone H4 | HIST1H4A | 4 | 31.1 | 11.367 |
| 111 | Q86YZ3 | Hornerin | HRNR | 13 | 13.2 | 282.39 |
| 112 | Q4G0P3 | Hydrocephalus-inducing protein homolog | HYDIN | 1 | 0.2 | 575.89 |
| 113 | P0DOX3 | Ig delta chain C region | IGHD | 2 | 5.9 | 56.224 |
| 114 | Q8NF17 | Ig gamma-3 chain C region | FLJ00385 | 7 | 37.3 | 56.11 |
| 115 | P01861 | Ig gamma-4 chain C region | IGHG4 | 3 | 24.7 | 43.831 |
| 116 | P01817 | Ig heavy chain V-II region MCE | IGHV2-70 | 1 | 26.1 | 13.231 |
| 117 | A0A5C2GIF5 | Ig kappa chain V-I region AG | IGKV1-39 | 1 | 26.2 | 11.809 |
| 118 | P01601 | Ig kappa chain V-I region HK101 | IGKV1-5 | 1 | 13.7 | 12.73 |
| 119 | Q5NV80 | Ig lambda chain V region 4A | V3-2 | 1 | 18.4 | 10.367 |
| 120 | A0A5C2GQT9 | Ig lambda chain V-I region NEWM | V1-13 | 1 | 96.4 | 11.508 |
| 121 | Q5NV89 | Ig lambda chain V-II region NEI | V1-7 | 1 | 8.1 | 10.286 |
| 122 | A0A5H1ZRQ7 | Ig lambda-7 chain C region | IGLC7 | 2 | 50.9 | 11.196 |
| 123 | P01871 | Ig mu chain C region | IGHM | 3 | 57.2 | 51.923 |
| 124 | Q9Y6R7 | IgGFc-binding protein | FCGBP | 12 | 4.3 | 572.01 |
| 125 | P01591 | Immunoglobulin J chain | IGJ | 8 | 71.1 | 18.098 |
| 126 | P08514 | Integrin alpha-Iib | ITGA2B | 9 | 10.7 | 113.38 |
| 127 | P05106 | Integrin beta-3 | ITGB3 | 9 | 12.8 | 87.057 |
| 128 | P19827 | Inter-alpha-trypsin inhibitor heavy chain H1 | ITIH1 | 3 | 4.7 | 101.39 |
| 129 | P19823 | Inter-alpha-trypsin inhibitor heavy chain H2 | ITIH2 | 8 | 8 | 106.46 |
| 130 | Q14624 | Inter-alpha-trypsin inhibitor heavy chain H4 | ITIH4 | 11 | 11.9 | 103.36 |
| 131 | Q9NPH3 | Interleukin-1 receptor accessory protein | IL1RAP | 1 | 1.6 | 65.418 |
| 132 | P14923 | Junction plakoglobin | JUP | 2 | 3.9 | 81.744 |
| 133 | P29622 | Kallistatin | SERPINA4 | 5 | 12.9 | 48.541 |
| 134 | P13645 | Keratin, type I cytoskeletal 10 | KRT10 | 27 | 58.4 | 58.826 |
| 135 | P02533 | Keratin, type I cytoskeletal 14 | KRT14 | 3 | 47.7 | 51.561 |
| 136 | P08779 | Keratin, type I cytoskeletal 16 | KRT16 | 11 | 50.7 | 51.267 |
| 137 | Q04695 | Keratin, type I cytoskeletal 17 | KRT17 | 3 | 33.8 | 48.105 |
| 138 | P35527 | Keratin, type I cytoskeletal 9 | KRT9 | 31 | 65.5 | 62.064 |
| 139 | P35908 | Keratin, type II cytoskeletal 2 epidermal | KRT2 | 1 | 68.1 | 65.432 |
| 140 | P13647 | Keratin, type II cytoskeletal 5 | KRT5 | 2 | 40.8 | 62.378 |
| 141 | P02538 | Keratin, type II cytoskeletal 6A | KRT6A | 2 | 39.9 | 60.044 |
| 142 | Q8N1N4 | Keratin, type II cytoskeletal 78 | KRT78 | 3 | 6.7 | 56.865 |
| 143 | Q5T749 | Keratinocyte proline-rich protein | KPRP | 4 | 7.3 | 64.135 |
| 144 | P01042 | Kininogen-1 | KNG1 | 7 | 13.8 | 71.957 |
| 145 | P18428 | Lipopolysaccharide-binding protein | LBP | 5 | 13.7 | 53.383 |
| 146 | P48740 | Mannan-binding lectin serine protease 1 | MASP1 | 5 | 22.5 | 79.246 |
| 147 | O00187 | Mannan-binding lectin serine protease 2 | MASP2 | 5 | 8.2 | 75.702 |
| 148 | P11226 | Mannose-binding protein C | MBL2 | 4 | 16.9 | 26.143 |
| 149 | Q6P4Q7 | Metal transporter CNNM4 | CNNM4 | 1 | 0.9 | 86.606 |
| 150 | P35579 | Myosin-9 | MYH9 | 5 | 2.8 | 226.53 |
| 151 | Q5T2W1 | Na(+)/H(+) exchange regulatory cofactor NHE-RF3 | PDZK1 | 1 | 3.1 | 57.129 |
| 152 | O76041 | Nebulette | NEBL | 1 | 0.8 | 116.45 |
| 153 | Q5VWK0 | Neuroblastoma breakpoint family member 6 | NBPF6 | 1 | 2.2 | 72.238 |
| 154 | P59665 | Neutrophil defensin 1 | DEFA1 | 2 | 19.1 | 10.201 |
| 155 | P62937 | Peptidyl-prolyl cis-trans isomerase A | PPIA | 1 | 5.5 | 18.012 |
| 156 | P32119 | Peroxiredoxin-2 | PRDX2 | 2 | 8.6 | 21.892 |
| 157 | P04180 | Phosphatidylcholine-sterol acyltransferase | LCAT | 2 | 5.7 | 49.577 |
| 158 | P42338 | Phosphatidylinositol 4,5-bisphosphate 3-kinase catalytic subunit beta isoform | PIK3CB | 1 | 0.7 | 122.76 |
| 159 | P03952 | Plasma kallikrein | KLKB1 | 3 | 5.5 | 71.342 |
| 160 | P05155 | Plasma protease C1 inhibitor | SERPING1 | 13 | 27.2 | 55.154 |
| 161 | P05154 | Plasma serine protease inhibitor | SERPINA5 | 1 | 2.7 | 45.674 |
| 162 | P00747 | Plasminogen | PLG | 22 | 36.9 | 90.568 |
| 163 | P02776 | Platelet factor 4 | PF4 | 4 | 43.6 | 10.845 |
| 164 | P13224 | Platelet glycoprotein Ib beta chain | GP1BB | 3 | 14.6 | 21.717 |
| 165 | Q13093 | Platelet-activating factor acetylhydrolase | PLA2G7 | 1 | 1.8 | 50.077 |
| 166 | P08567 | Pleckstrin | PLEK | 1 | 3.7 | 40.124 |
| 167 | P01833 | Polymeric immunoglobulin receptor | PIGR | 10 | 15.7 | 83.283 |
| 168 | P0CG48 | Polyubiquitin-C | UBC | 1 | 11.8 | 77.038 |
| 169 | Q9UHG3 | Prenylcysteine oxidase 1 | PCYOX1 | 4 | 8.3 | 56.639 |
| 170 | P07737 | Profilin-1 | PFN1 | 1 | 10 | 15.054 |
| 171 | P27918 | Properdin | CFP | 14 | 35.4 | 51.276 |
| 172 | P02760 | Protein AMBP | AMBP | 6 | 22.4 | 38.999 |
| 173 | P49757 | Protein numb homolog | NUMB | 1 | 1.7 | 70.803 |
| 174 | P05109 | Protein S100-A8 | S100A8 | 1 | 11.8 | 10.834 |
| 175 | P06702 | Protein S100-A9 | S100A9 | 1 | 11.4 | 13.242 |
| 176 | Q8TF72 | Protein Shroom3 | SHROOM3 | 1 | 0.5 | 216.85 |
| 177 | Q92954 | Proteoglycan 4 | PRG4 | 1 | 1.2 | 151.06 |
| 178 | P00734 | Prothrombin | F2 | 8 | 20.6 | 70.036 |
| 179 | P14618 | Pyruvate kinase PKM | PKM | 2 | 5.1 | 57.936 |
| 180 | Q15404 | Ras suppressor protein 1 | RSU1 | 1 | 6.5 | 31.54 |
| 181 | P20340 | Ras-related protein Rab-6A | RAB6A | 1 | 5.3 | 23.593 |
| 182 | P61224 | Ras-related protein Rap-1b | RAP1B | 5 | 32.1 | 20.825 |
| 183 | Q13103 | Secreted phosphoprotein 24 | SPP2 | 1 | 5.7 | 24.337 |
| 184 | P49908 | Selenoprotein P | SEPP1 | 3 | 8.1 | 43.173 |
| 185 | P02787 | Serotransferrin | TF | 22 | 36.4 | 77.049 |
| 186 | P02768 | Serum albumin | ALB | 44 | 63.7 | 69.366 |
| 187 | P0DJI8 | Serum amyloid A-1 protein | SAA1 | 1 | 23.8 | 13.546 |
| 188 | A0A096LPE2 | Serum amyloid A-4 protein | SAA2-SAA4 | 2 | 13.5 | 23.353 |
| 189 | P02743 | Serum amyloid P-component | APCS | 5 | 22.4 | 25.387 |
| 190 | O95810 | Serum deprivation-response protein | SDPR | 1 | 2.6 | 47.173 |
| 191 | P27169 | Serum paraoxonase/arylesterase 1 | PON1 | 10 | 36.9 | 39.731 |
| 192 | I1VSB5 | Sialic acid-binding Ig-like lectin 16 | SIGLEC16 | 1 | 1.6 | 47.251 |
| 193 | Q9H254 | Spectrin beta chain, non-erythrocytic 4 | SPTBN4 | 1 | 0.4 | 288.98 |
| 194 | P24821 | Tenascin | TNC | 11 | 6.9 | 240.85 |
| 195 | P10599 | Thioredoxin | TXN | 2 | 21 | 11.737 |
| 196 | P07996 | Thrombospondin-1 | THBS1 | 24 | 26.6 | 129.38 |
| 197 | P35443 | Thrombospondin-4 | THBS4 | 4 | 6.1 | 105.87 |
| 198 | P02786 | Transferrin receptor protein 1 | TFRC | 1 | 1.8 | 84.87 |
| 199 | P02766 | Transthyretin | TTR | 5 | 40.1 | 15.887 |
| 200 | P35030 | Trypsin-3 | PRSS3 | 1 | 4.3 | 32.528 |
| 201 | P68363 | Tubulin alpha-1B chain | TUBA1B | 1 | 1.6 | 50.151 |
| 202 | P59282 | Tubulin polymerization-promoting protein family member 2 | TPPP2 | 1 | 4.7 | 18.502 |
| 203 | O75643 | U5 small nuclear ribonucleoprotein 200 kDa helicase | SNRNP200 | 1 | 0.4 | 244.5 |
| 204 | Q9P1Q0 | Vacuolar protein sorting-associated protein 54 | VPS54 | 2 | 1.7 | 110.59 |
| 205 | P18206 | Vinculin | VCL | 7 | 7 | 123.8 |
| 206 | P02774 | Vitamin D-binding protein | GC | 6 | 14.8 | 52.917 |
| 207 | P07225 | Vitamin K-dependent protein S | PROS1 | 25 | 36.1 | 75.122 |
| 208 | P04004 | Vitronectin | VTN | 14 | 31.2 | 54.305 |
| 209 | P04275 | von Willebrand factor | VWF | 2 | 27.2 | 309.26 |
| 210 | Q99592 | Zinc finger and BTB domain-containing protein 18 | ZBTB18 | 1 | 1.3 | 58.354 |
| 211 | P25311 | Zinc-alpha-2-glycoprotein | AZGP1 | 2 | 9.1 | 34.258 |


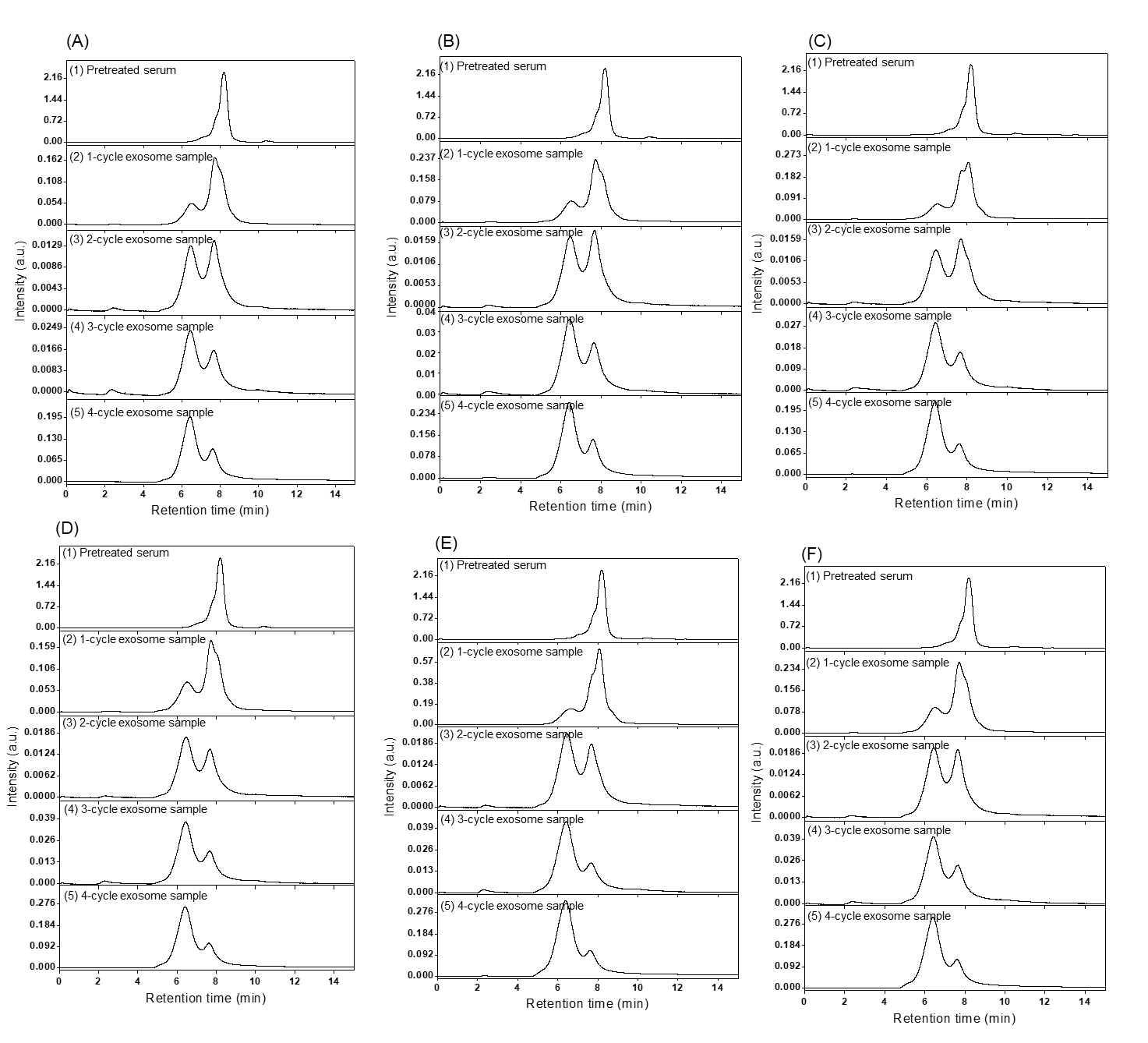


**Figure S1.** SEC chromatograms obtained with UV detection at 280 nm for pretreated serum and exosome samples enriched through 1-cycle, 2-cycle, 3-cycle, and 4-cycle steps using (A) PEG 8,000, (B) PEG 10,000, (C) PEG 12,000, (D) PEG 20,000, (E) PEG 35,000 and (F) ExoQuick. The PEG percentage used for the enrichment was 3.3 (w/v)%.

**
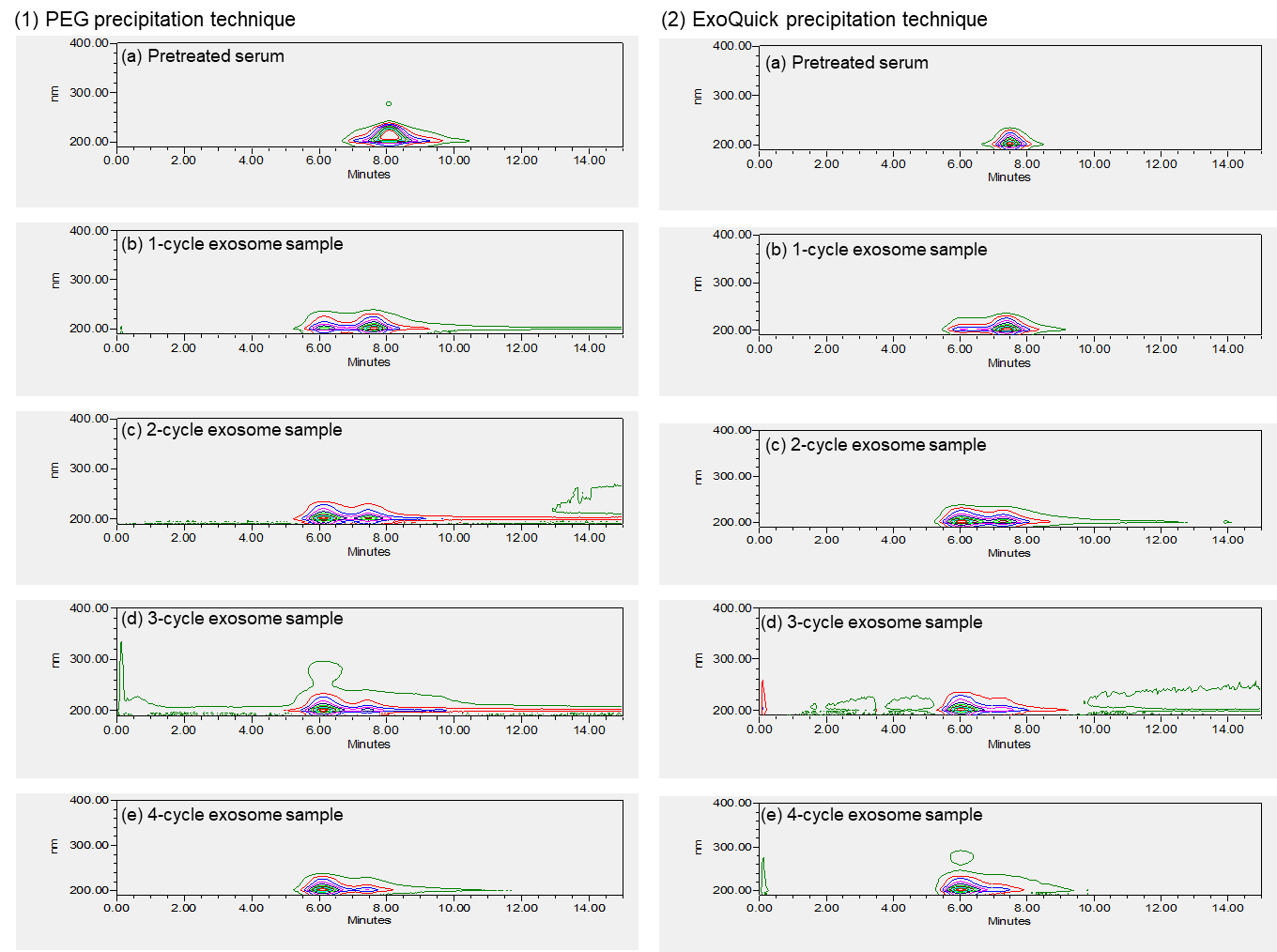
**

**Figure S2.** UV contour spectra obtained with PDA detection for pretreated serum and exosome samples enriched through 1-cycle, 2-cycle, 3-cycle, and 4-cycle steps using (A) PEG 10,000 and (B) ExoQuick. The percentage of PEG 10,000 used for the enrichment was 3.3 (w/v)%.

**Figure S3.** SEC chromatograms obtained with UV detection at 280 nm for (A) bovine serum albumin and (B) human immunoglobulin G. The samples were prepared in PBS at a concentration of 1000 ppm.


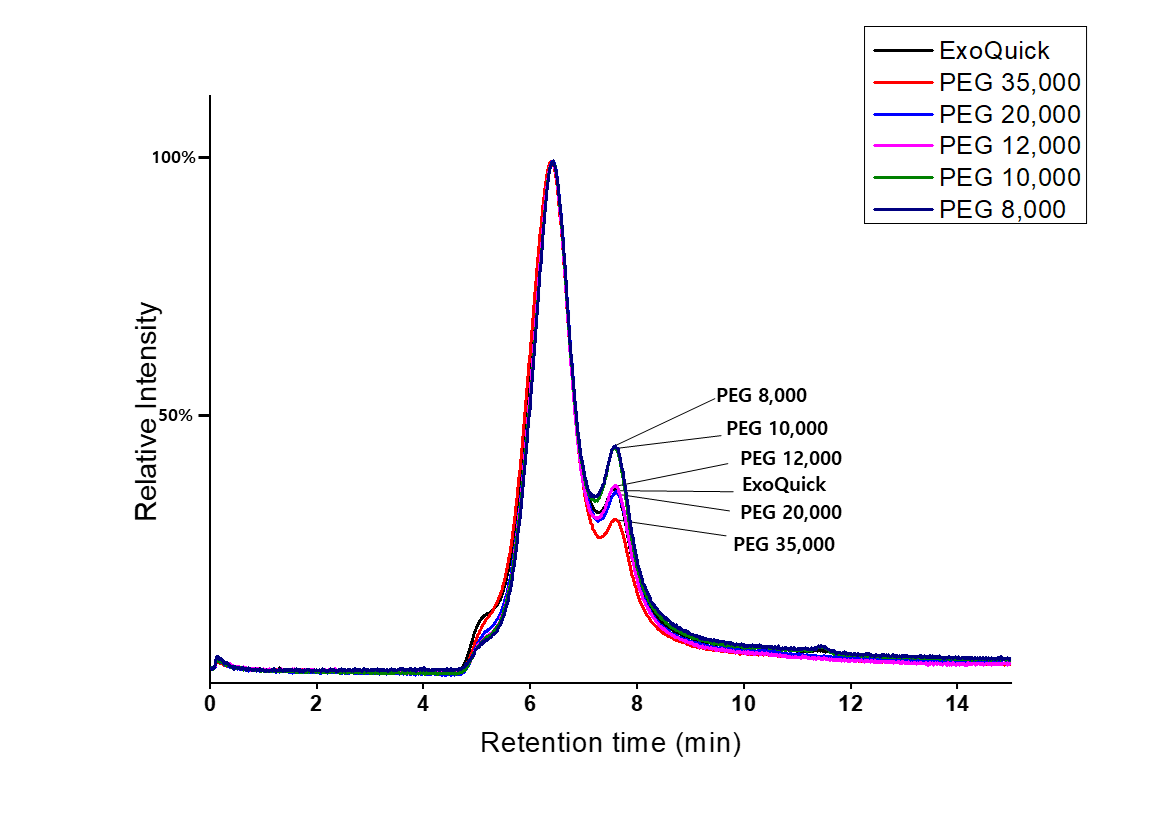


**Figure S4.** SEC chromatograms obtained with UV detection at 280 nm for the exosome enriched through 4-cycle step using PEG 8,000, 10,000, 12,000, 20,000, 35,000 and ExoQuick. The exosome peak around 6 min was normalized to 100% across all samples to maintain consistency in evaluating the protein peak around 8 min. The PEG concentration used for the enrichment was 3.3 (w/v)%.

**Figure S5.** MALDI-TOF MS spectra of (A) PEG 8,000, (B) PEG 10,000, (C) ExoQuick, (D) PEG 12,000, (E) PEG 20,000, and (F) PEG 35,000.

**Figure S6.** SEC chromatograms obtained with UV detection at 280 nm for pretreated serum and exosome samples enriched through 1-cycle, 2-cycle, 3-cycle, and 4-cycle steps using different PEG 10,000 concentrations of (A) 1.7, (B) 3.3, (C) 6.6, (D) 11.0, and (E) 16.5 (w/v)%.

**Figure S7.** SEC chromatograms obtained with UV detection at 280 nm for the 4-cycle exosome samples enriched using different PEG 10,000 concentrations of (A) 1.7, (B) 3.3, (C) 6.6, (D) 11.0, and (E) 16.5 (w/v)%.


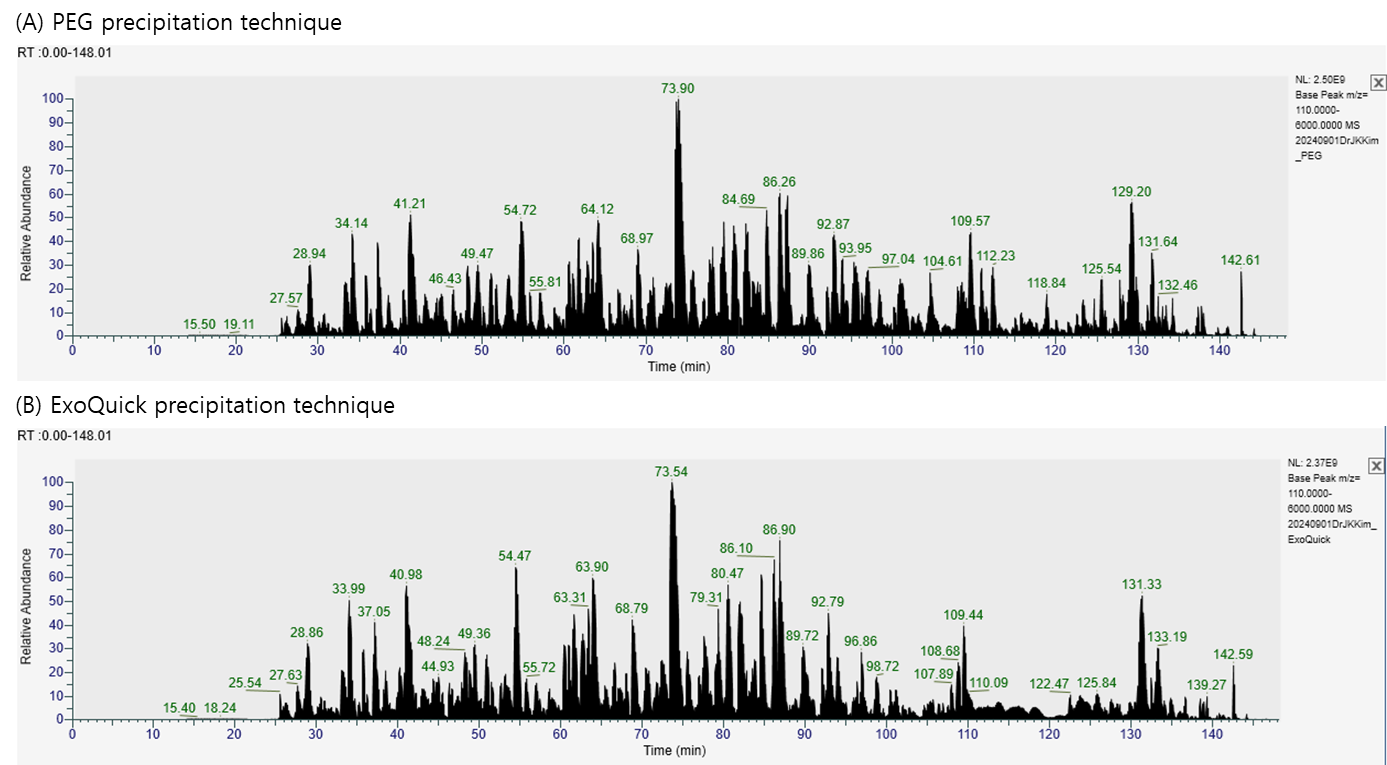


**Figure S8.** Typical nano-LC-MS/MS chromatograms of exosome-enriched samples isolated using (A) PEG precipitation and (B) ExoQuick precipitation techniques. The chromatograms illustrate the proteomic profiles obtained from each enrichment method.
